# A genome-wide identification and analysis of the basic helix-loop-helix transcription factors in the ponerine ant, *Harpegnathos saltator*

**DOI:** 10.1186/1471-2148-12-165

**Published:** 2012-08-31

**Authors:** Ake Liu, Yong Wang, Chunwang Dang, Debao Zhang, Huifang Song, Qin Yao, Keping Chen

**Affiliations:** 1 Institute of Life Sciences, Jiangsu University, 301 Xuefu Road, Zhenjiang, 212013, P. R., China; 2 School of Food and Biological Engineering, Jiangsu University, 301 Xuefu Road, Zhenjiang 212013, P. R., China

**Keywords:** Basic helix-loop-helix, Transcription factor, Harpegnathos saltator, Phylogenetic analysis, Orthologous family, Blast search

## Abstract

**Background:**

The basic helix-loop-helix (bHLH) transcription factors and their homologs form a superfamily that plays essential roles in transcriptional networks of multiple developmental processes. bHLH family members have been identified in over 20 organisms, including fruit fly, zebrafish, human and mouse.

**Result:**

In this study, we conducted a genome-wide survey for bHLH sequences, and identified 57 bHLH sequences encoded in complete genome sequence of the ponerine ant, *Harpegnathos saltator*. Phylogenetic analysis of the bHLH domain sequences classified these genes into 38 bHLH families with 23, 14, 10, 1, 8 and 1 members in group A, B, C, D, E and F, respectively. The number of PabHLHs (ponerine ant bHLHs) with introns is higher than many other insect species, and they are found to have introns with average lengths only inferior to those of pea aphid. In addition, two *H. saltator* bHLHs named *PaCrp1* and *PaSide* locate on two separate contigs in the genome.

**Conclusions:**

A putative full set of PabHLH genes is comparable with other insect species and genes encoding Oligo, MyoRb and Figα were not found in genomes of all insect species of which bHLH family members have been identified. Moreover, in-family phylogenetic analyses indicate that the PabHLH genes are more closely related with *Apis mellifera* than others. The present study will serve as a solid foundation for further investigations into the structure and function of bHLH proteins in the regulation of *H. saltator* development.

## Background

Since the first basic helix-loop-helix (bHLH) motif with DNA-binding and dimerization capabilities was reported [1], numerous bHLH proteins have been found to be intimately involved in the regulation of a wide range of developmental processes, including neurogenesis, myogenesis, hematopoiesis, sex determination, gut development, cell differentiation and proliferation, as well as other essential processes in organisms ranging from yeast to humans [2,3]. Hence, it is crucial that we understand the relationship of the various bHLH members, and be able to classify them into well-defined categories. These transcription factors, having a signature bHLH structural motif of approximately 60 amino acids and 19 highly conserved amino acids, consist of a basic region followed by two α-helices separated by a loop (HLH) region of variable length [2]. Working as a DNA-binding domain, the two basic domains dimerize to create a DNA interaction surface that recognizes the consensus hexanucleotide sequence, while the HLH domain interacts with other bHLH proteins to form homodimers or heterodimers between different bHLH family members [4,5].

In 1997, a phylogenetic analysis based on 122 bHLH sequences resulted in a natural classification of different bHLH transcription factors into four monophyletic protein groups named A, B, C and D in an attempt to functionally classify bHLH proteins [6]. Since more bHLH proteins had been identified in animals, plants and fungi, 44 orthologous families and six higher-order groups had been defined based on phylogenetic analyses to then available bHLH proteins [3,6-8]. In addition, after the revision of Simionato et al. in 2007, animal bHLH proteins are classified into 45 families, among which 22, 12, 7, 1, 2 and 1 families are included in high order groups A, B, C, D, E and F, respectively [9]. Briefly, groups A and B bHLH proteins are inclined to bind core DNA sequences typical of E boxes (CANNTG), in which group A recognizes and binds CACCTG or CAGCTG and group B recognizes and binds CACGTG or CATGTTG. Group A bHLH proteins mainly regulate neurogenesis, myogenesis and mesoderm formation, while group B ones mainly regulate cell proliferation and differentiation, sterol metabolism and adipocyte formation, and expression of glucose-responsive genes. Group C proteins, complex molecules with one or two PAS domains following the bHLH motif, tend to bind the core sequence of ACGTG or GCGTG. They are responsible for the regulation of midline and tracheal development, circadian rhythms, and for the activation of gene transcription in response to environmental toxins. Group D proteins correspond to bHLH proteins that are unable to bind DNA due to lack of a basic domain and act as antagonists of group A proteins. Group E proteins, mainly regulating embryonic segmentation, somitogenesis and organogenesis, bind preferentially to sequences referred to as N boxes (CACGCG or CACGAG) and usually contain two characteristic domains named “Orange” and “WRPW” peptide in the carboxyl terminus. Group F proteins have the COE domain which has an additional domain involved in both dimerization and DNA binding. It has only one family, and mainly regulates head development and formation of olfactory sensory neurons [7,10].

Due to the pivotal regulatory functions of bHLH proteins displaying in various organisms and the completion of genome sequencing projects for an increased number of organisms, it would be desirable to have a more refined classification scheme of the various types of bHLH motifs, as well as a better understanding of their evolutionary relationships both within and among species. Large numbers of bHLH family members have been the subject of several studies targeting the identification of their full complement encoded by genomes completely sequenced. The putative full set of genes encoding bHLH proteins has been reported to be 8 bHLH genes in *Saccharomyces cerevisiae*, 16 in *Amphimedon queenslandica*, 33 in *Hydra magnipapillata*, 42 in *Caenorhabditis elegans*, 46 in *Ciona intestinalis*, 50 in *Strongylocentrotus purpuratus*, 50 in *Tribolium castaneum*, 51 in *Apis mellifera*, 52 in *Bombyx mori*, 54 in *Acyrthosiphon pisum*, 57 in *Daphnia pulex*, 59 in *Drosophila melanogaster*, 63 in *Lottia gigantea*, 64 in *Capitella* sp 1, 68 in *Nematodtella vectensis*, 70 in *Acropora digitifera*, 78 in *Branchiostoma floridae*, 87 in *Tetraodon nigroviridis*, 104 in *Gallus gallus*, 107 in *Ailuropoda melanoleuca*, 114 in *Mus musculus*, 114 in *Rattus norvegicus*, 118 in *Homo sapiens*, 139 in *Danio rerio*, 162 in *Arabidopsis thaliana*, and 167 in *Oryza sativa*[9-21].

Ponerine ant, *Harpegnathos saltator* (Jerdon, 1851), has recently been introduced as a model organism for studying the relationship between stress resistance and longevity of eusocial insects, as well as the role of epigenetics in behavior, aging, and development. Several studies have recently been conducted to elucidate the developmental processes that result in its particular characters [22,23]. However, the *H. saltator* bHLH proteins have not yet been studied and characterized in detail. The *H. saltator* genome is the first ant genome having been sequenced. The draft *H. saltator* genome assembly sequenced using the Illumina Genome Analyzer platform was submitted by the Beijing Genomics Institute –Shenzhen in August 2010. Moreover, the *H. saltator* draft genomic assemblies reached scaffold N50 with a size of ~600 kb and covered more than 90% of the genomes [22].

The comprehensive identification of bHLH protein members encoded in the *H. saltator* genome would facilitate experimental studies on biological functions of bHLH proteins in the regulation of *H. saltator* development as well as evolutionary analyses to the diversification of insect bHLH genes. In this study, tblastn searches against *H. saltator* genome sequence database was conducted using both amino acid sequences of 59 *Drosophila melanogaster* bHLH (DmbHLH) motifs [17] and the 45 representative bHLH families (Additional file 1) [7] to retrieve candidate bHLH members. Subsequent examination and phylogenetic analysis enabled us to identify the putative full set of bHLH members encoded in *H. saltator* and to define orthologous families with sufficient confidence. The obtained results are helpful for further investigations into the structure and function of bHLH proteins in the regulation of *H. saltator* development.

## Results and discussions

### Identification of PabHLH members

**Figure 1:**
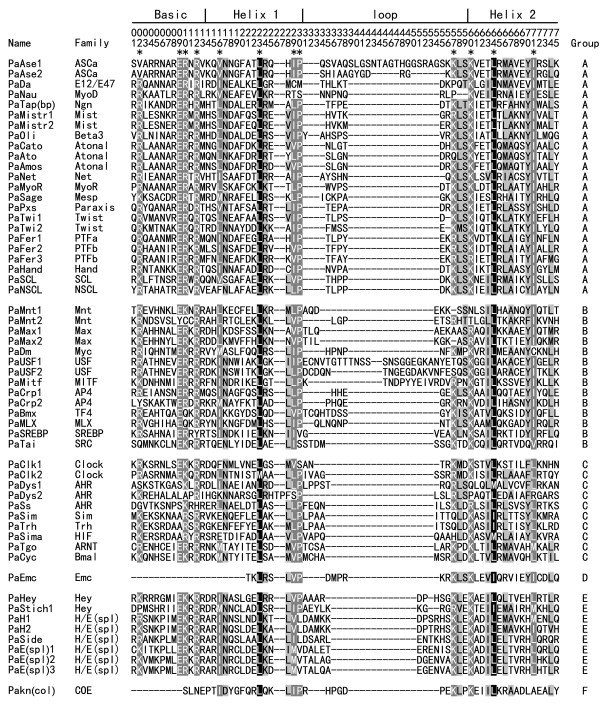
**Multiple sequence aligment of bHLH motifs of the 57 PabHLH sequences.** The scheme at top illustrates the locations and boundaries of the basic, helix 1, loop and helix 2 regions within the bHLH domain following that of Ferre-D’Amare et al. ( 1993). The numbers below the scheme (1 to 75) present the position within the bHLH motif as defined in this study. The shading of the alignment indicates identical residues in black, conserved residues in dark gray and similar residues in light gray. Highly conserved sites are indicated with asterisks on the top. Strigula denote gaps. The family names and high-order groups have been organized according to Table 1 of Ledent et al. ( 2002).

**Figure 2:**
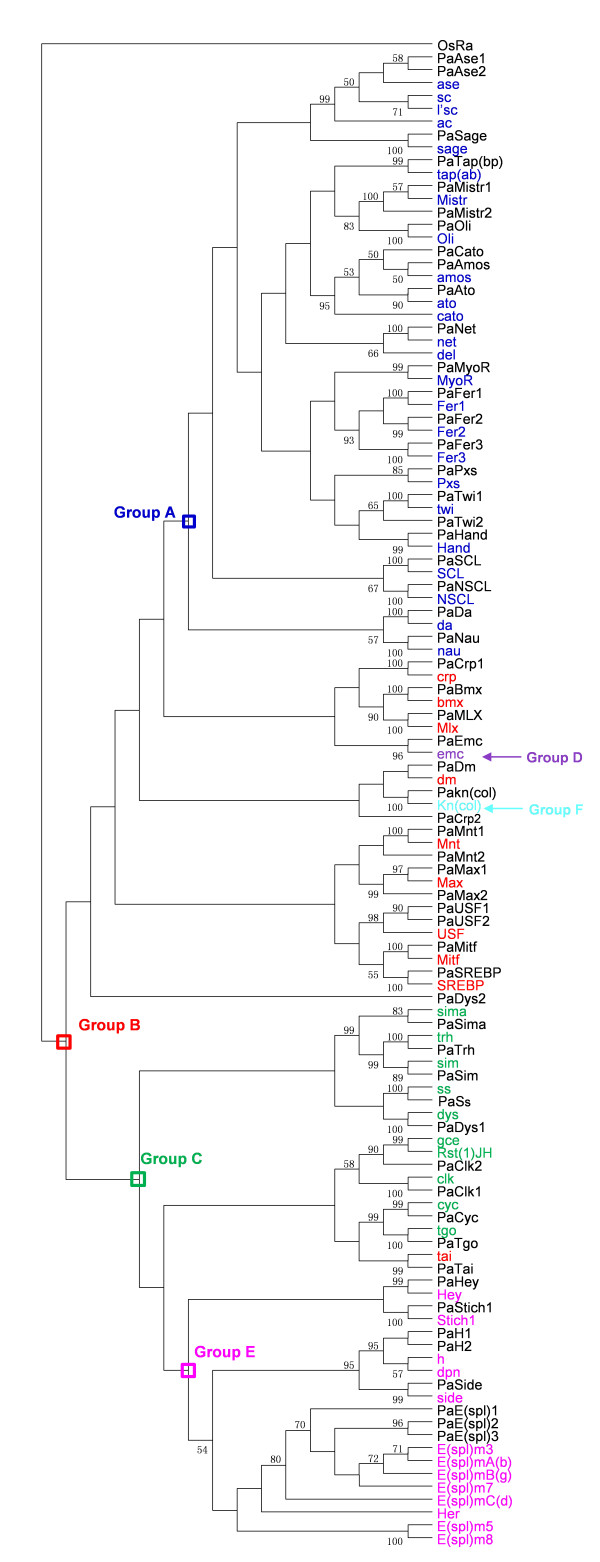
**Neighbor-joining phylogenetic tree of 57 PabHLH members with 59 *Drosophila melanogaster *bHLH members.** The NJ tree summarizes the evolutionary relationship between the PabHLHs and DmbHLHs, which has been rooted using OsRa (a rice bHLH motif sequence of R family) as outgroup. This tree is based on a multiple alignment that includes 59 bHLH sequences of *D. melanogaster* and 57 PabHLH members. For simplicity, branch lengths of the tree are not proportional to distances between sequences. Only bootstrap values more than 50 are shown. The higher-order group labels are in accordance with Ledent et al. ( 2002).

The tblastn searches, intron analysis, manual checking of the 19 conserved amino acid sites, and sequence alignment reveal that there are 57 bHLH members in *H. saltator* (Additional file 2). The alignment of all 57 PabHLH members is shown in Figure 1, and the phylogenetic tree generated utilizing amino acids of 57 PabHLH motifs and 59 DmbHLH motifs is illustrated in Figure 2. Both figures demonstrate that there are 23, 14, 10, 1, 8 and 1 PabHLH members in group A, B, C, D, E and F, respectively. It can be seen from Figure 1 that sites 23 and 64 of the bHLH motif are the most conserved sites among all PabHLH motifs. Besides, other ten sites, marked with asterisks on top of Figure 1, are also highly conserved. Additionally, Figure 1 indicates that one PabHLH motif *PaDys2* has quite special amino acids. Whereas the alignments of all identified bHLH motifs in other insect species have no gaps in the basic and helix 1 regions and only one major gap in the loop region [13,14], *PaDys2* has two additional amino acids (T and P) in helix 1 region. The existence of these amino acids has created an additional gap among aligned PabHLH motifs (Figure 1), indicating certain difference between *H. saltator* and other insect species. From Figure 2, we found two cases, like *PaAse1* and *PaAse2* which can form a monophyletic clade with *ase* from fruit fly, that the two PabHLHs group together with high statistical support to the exclusion of any other sequences and are often orthologs of a single fruit fly motif. This may reveal relatively recent duplications specific to the *H. saltator*. Besides these characters on Figure 2, two PabHLHs, *PaH1* and *PaH2*, can be related to two *D. melanogaster* families of orthologs. This may indicate that the diversity of those bHLHs found in both species occurred before the generation of duplications.

During our analyses, one sequence “RREIANSNERRRMQSINAGFQSLRSLLPHHEGEKLSKVCIV” (contig number: AEAC01009287.1, coding region from 29454 to 29576) was found to have high similarity with AP4 family. However, the immediately following codon is a stop codon. We consider that it could be a pseudogene or the sequence with a nonsense mutation. But it is also possible that the protein sequence coded by this gene was not accurately predicted.

Based on bootstrap supports provided by the in-group phylogenetic analyses, the identified PabHLH motifs were subdivided into corresponding bHLH families and named according to nomenclature used by DmbHLH sequences. Although a new nomenclature for bHLH proteins has been proposed recently [24], we adopted nomenclature used in *D. melanogaster* for facilitating further studies on structural and functional comparison with *D. melanogaster*. In case that one DmbHLH sequence has two or more *H. saltator* homologues, we use ‘1’, ‘2’ and ‘3’ etc. to number them. For instance, two homologues of the *D. melanogaster Mistr* and *USF* genes were found in *H. saltator*, respectively. Therefore, these PabHLH genes were named *PaMistr1* and *PaMistr2*, *PaUSF1*, and *PaUSF2*, respectively. Names of 57 PabHLHs in accordance with their corresponding *D. melanogaster* homologues are listed in Table 1.

**Table 1:** **A complete list of bHLH genes from *Harpegnathos saltator***

**No.**	**Gene name**	**Family**	**Fruit fly homolog**	**Bootstrap values**			**Protein accession No.**
				**NJ**	**MP**	**ML**	
01	PaAse1*	ASCa	*ase*	99	98	97	EFN85365.1
02	*PaAse2*	ASCa	*ase*	44	33	61	EFN85366.1
03	*PaDa*	E12/E47	*da*	100	100	92	EFN82122.1
04	*PaNau*	MyoD	*nau*	100	99	57	EFN79255.1
05	*PaTap(bp)*	Ngn	*tap (bp)*	99	97	84	EFN75119.1
06	*PaMistr1*	Mist	*Mistr*	100	100	70	EFN88257.1
07	*PaMistr2*	Mist	*Mistr*	100	97	65	EFN75769.1
08	*PaOli*	Beta3	*Oli*	100	100	59	EFN75891.1
09	*PaCato*	Atonal	*cato*	48	43	55	EFN82083.1
10	*PaAto*	Atonal	*ato*	98	94	75	Not available
11	*PaAmos*	Atonal	*amos*	84	65	50	EFN82082.1
12	*PaNet*	Net	*net*	100	100	63	Not available
13	*PaMyoR*	MyoRa	*MyoR*	99	97	64	EFN78165.1
14	*PaSage*	Mesp	*sage*	100	100	94	Not available
15	*PaPxs*	Paraxis	*Pxs*	93	77	88	Not available
16	*PaTwi1*	Twist	*twi*	100	99	83	EFN77900.1
17	*PaTwi2*	Twist	*twi*	63	43	73	EFN77901.1
18	*PaFer1*	PTFa	*Fer1*	100	78	63	EFN75358.1
19	*PaFer2*	PTFb	*Fer2*	99	95	53	EFN80609.1
20	*PaFer3*	PTFb	*Fer3*	100	100	88	EFN77527.1
21	*PaHand*	Hand	*Hand*	99	95	50	EFN90007.1
22	*PaSCL*	SCL	*SCL*	100	100	59	EFN82626.1
23	*PaNSCL*	NSCL	*NSCL*	100	100	67	EFN83537.1
24	*PaMnt1*	Mnt	*Mnt*	100	100	88	EFN84151.1
25	PaMnt2*	Mnt	*Mnt*	95	52	66	Not available
26	*PaMax1*	Max	*Max*	100	98	86	EFN76634.1
27	*PaMax2*	Max	*max*	96	80	73	EFN83237.1
28	*PaDm*	Myc	*dm*	82	76	68	EFN89178.1
29	*PaUSF1*	USF	*USF*	100	89	95	EFN78146.1
30	*PaUSF2*	USF	*USF*	99	94	95	EFN76085.1
31	*PaMitf*	MITF	*Mitf*	100	100	80	EFN77564.1
32	*PaCrp1*	AP4	*Crp*	100	100	94	Not available
33	PaCrp2*	AP4	*Crp*	100	100	70	Not available
34	*PaBmx*	TF4	*bmx*	100	96	89	EFN84400.1
35	*PaMLX*	MLX	*MLX*	100	100	97	EFN77615.1
36	*PaSREBP*	SREBP	*SREBP*	100	100	70	EFN85492.1
37	*PaTai*	SRC	*tai*	100	99	82	EFN80872.1
38	*PaClk1*	Clock	*clk*	100	99	94	EFN76178.1
39	PaClk2*	Clock	*clk*	100	100	90	Not available
40	*PaDys1*	AHR	*dys*	100	100	82	Not available
41	PaDys2*	AHR	*dys*	58	70	n/m	Not available
42	*PaSs*	AHR	*ss*	100	100	83	EFN80844.1
43	*PaSim*	Sim	*sim*	88	92	63	EFN79346.1
44	*PaTrh*	Trh	*trh*	100	94	98	EFN81642.1
45	*PaSima*	HIF	*sima*	95	94	94	EFN75729.1
46	*PaTgo*	ARNT	*tgo*	100	100	99	Not available
47	*PaCyc*	Bmal	*cyc*	99	83	68	EFN88377.1
48	*PaEmc*	Emc	*emc*	99	83	74	EFN83186.1
49	*PaHey*	Hey	*Hey*	98	45	n/m*	EFN83075.1
50	*PaStich1*	Hey	*Stich1*	100	99	94	EFN89077.1
51	*PaH1*	H/E(spl)	*h*	69	70	64	EFN78278.1
52	*PaH2*	H/E(spl)	*h*	n/m	47	n/m*	EFN78273.1
53	*PaSide*	H/E(spl)	*side*	99	100	94	EFN79220.1
54	PaE(spl)1*	H/E(spl)	*E(spl) mC(d)*	98	50	n/m*	EFN87932.1
55	PaE(spl)2*	H/E(spl)	? - ortholog of *AmE(spl)2*	80	67	52	EFN87924.1
56	PaE(spl)3*	H/E(spl)	? - ortholog of AmE(spl)3	100	96	99	EFN87930.1
57	*Pakn(col)*	COE	*kn (col)*	100	100	92	EFN79194.1

We named PabHLH genes according to their *D. melanogaster* homologues. Bootstrap values were obtained from in-group phylogenetic analyses with *D. melanogaster* or *A. mellifera* bHLH motif sequences using NJ, MP, and ML algorithms, respectively. OsRa (the rice bHLH motif sequence of R family) was used as the outgroup in each constructed tree. n/m means that a *H. saltator* bHLH does not form a monophyletic group with any other single bHLH motif sequence. n/m* means that a *H. saltator* bHLH does not form a monophyletic clade with any specific bHLH motif sequence but forms a monophyletic clade with other bHLH proteins of the same family. * means that orthology of the gene was defined through in-group phylogenetic analyses with bHLH orthologs from *A. mellifera*.

### Identification of orthologous families

Orthologous genes in two or more organisms are those that evolved by vertical descent from the same gene in the last common ancestor [25]. Ortholog identification has much uncertainty because of the lack of absolute criterion that can be applied to decide whether two genes are orthologous or not [7]. Nevertheless, in our previous studies [13,14], in-group phylogenetic analysis was adopted to identify homologues for the unknown sequences that would form a monophyletic clade among themselves. Therefore, a more certain standard based on the criterion used by Ledent et al. was used in this study. That is, we defined bHLH families of orthologs as monophyletic groups which include sequences of a known family and whose monophyly is consistent with the different phylogenetic algorithms and supported by bootstrap values superior to 50 [7,9,17].

**Figure 3:**
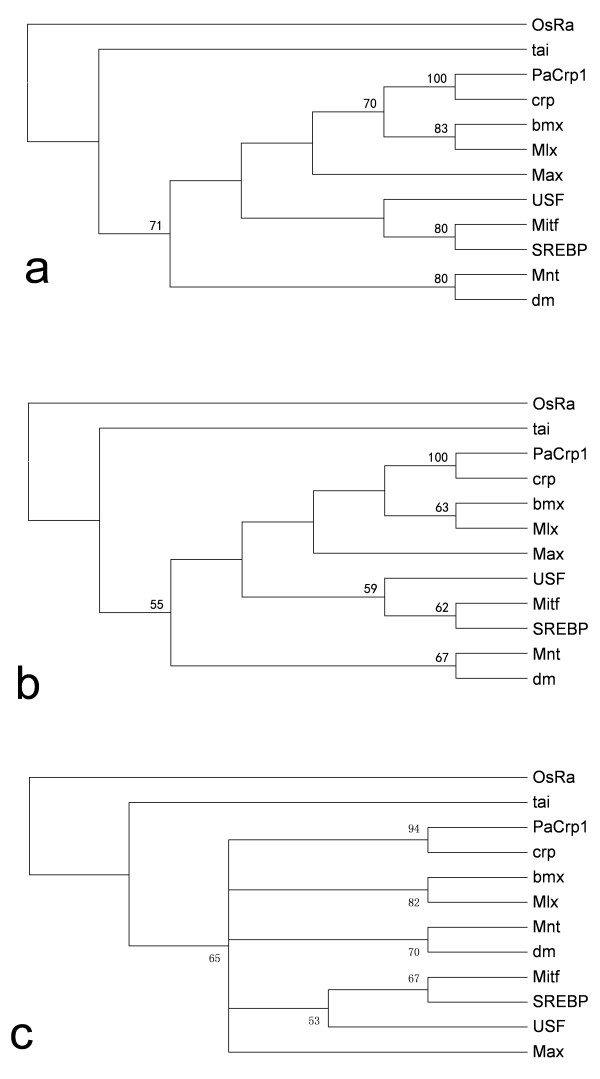
**In-group phylogenetic analyses of *PaCrp1*.** (**a**), (**b**), and (**c**) are NJ, MP, and ML trees, respectively, constructed with one *H. saltator* bHLH member (*PaCrp1*) and ten group B bHLH members from *D. melanogaster*. In all trees, OsRa was used as the outgroup.

We have performed in-group phylogenetic analysis to each of the 57 identified bHLHs, which enabled us to allocate all the identified PabHLHs to defined evolutionary conserved groups of orthology. Figure 3, as an example here, shows distance neighbour-joining (NJ), maximum parsimony (MP), and maximum likelihood (ML) phylogenetic trees constructed with one PabHLH member (*PaCrp1*) and 10 group B bHLH members from *D. melanogaster*. *PaCrp1* formed monophyletic clade with crp (cropped) sequence of *D. melanogaster* with bootstrap values of 100, 100 and 94 in NJ, MP and ML phylogenetic trees, respectively. *PaCrp1* was therefore considered as an ortholog of fruit fly *crp*. Similarly, in-group phylogenetic analysis was conducted to each of the identified PabHLH members. All the bootstrap values of constructed NJ, MP and ML trees for each of the identified PabHLH members were listed in Table 1 without displaying the correspondent constructed trees. The majority of these bHLHs could be clearly allocated to the families defined according to bootstrap values of in-group phylogenetic trees. Nevertheless, eight PabHLHs (a significant proportion, about 14%) could not be confidently allocated to the defined families by our phylogenetic analysis with DmbHLHs. They were used to construct trees with *Apis mellifera* bHLHs (AmbHLH) using the same methods mentioned above. Table 1 showed that orthology of PabHLHs with fruit fly or honey bee bHLHs could be divided into the following categories.

Firstly, among all the 57 PabHLH members, there are 44 bHLH members of which all the bootstrap values ranged from 50 to 100 in constructed NJ, MP and ML trees. Namely, they are *PaDa*, *PaNau*, *PaTap(bp)*, *PaMistr1*, *PaMistr2*, *PaOli*, *PaAto*, *PaAmos*, *PaNet*, *PaMyoR*, *PaSage*, *PaPxs*, *PaTwi1*, *PaFer1*, *PaFer2*, *PaFer3*, *PaHand*, *PaSCL*, *PaNSCL*, *PaMnt1*, *PaMax1*, *PaMax2*, *PaDm*, *PaUSF1*, *PaUSF2*, *PaMitf*, *PaCrp1*, *PaBmx*, *PaMLX*, *PaSREBP*, *PaTai*, *PaClk1*, *PaDys1*, *PaSs*, *PaSim*, *PaTrh*, *PaSima*, *PaTgo*, *PaCyc*, *PaEmc*, *PaStich1*, *PaH1*, *PaSide* and *PaKn(col)*. All these bootstrap values were no lower than the set criterion (50) and thus resulted in assignment of corresponding *D. melanogaster* homologues for them with sufficient bootstrap support (Table 2).

**Table 2:** Coding regions, intron location and length of 57 PabHLH motifs

**Family**	**Gene name**	**Genomic coding sequence(s)**			**Intron (location,length)**	**Group**
		**Contig no.**	**Frame**	**Coding region(s)**		
ASCa	*PaAse1*	AEAC01008798.1	+3	9033-9242		A
ASCa	*PaAse2*	AEAC01008800.1	+2	11015-11191		A
E12/E47	*PaDa*	AEAC01014068.1	+3	28419-28447	Basic: 805 bp	A
			+1	29253-29385		
MyoD	*PaNau*	AEAC01019196.1	−2	74906-74862	Helix 1: 1290bp	A
			−2	73571-73461		
Ngn	*PaTap(bp)*	AEAC01026729.1	−1	32156-31998		A
Mist	*PaMistr1*	AEAC01003460.1	−2	3518-3456	Helix 1: 100bp	A
			−3	3355-3260		
Mist	*PaMistr2*	AEAC01025732.1	−1	1835-1773	Helix 1: 788bp	A
			−3	984-889		
Beta3	*PaOli*	AEAC01025379.1	+2	21989-22153		A
Atonal	*PaCato*	AEAC01014266.1	−1	26350-26192		A
Atonal	*PaAto*	AEAC01015400.1	+2	188-346		A
Atonal	*PaAmos*	AEAC01014265.1	−1	11834-11676		A
Net	*PaNet*	AEAC01000094.1	+1	43-201		A
MyoRa	*PaMyoR*	AEAC01020981.1	−1	91681-91523		A
Mesp	*PaSage*	AEAC01024430.1	−2	277-116		A
Paraxis	*PaPxs*	AEAC01022136.1	+3	27672-27715	Helix 1: 560bp	A
			+2	28276-28390		
Twist	*PaTwi1*	AEAC01021518.1	−3	26828-26673		A
Twist	*PaTwi2*	AEAC01021518.1	−3	48350-48195		A
PTFa	*PaFer1*	AEAC01026349.1	+1	45976-46134		A
PTFb	*PaFer2*	AEAC01016863.1	−3	13162-13119	Helix 1: 410bp	A
			−2	12708-12594		
PTFb	*PaFer3*	AEAC01022136.1	+2	110825-110905	Helix 1: 2060bp	A
			+1	112966-113043		
Hand	*PaHand*	AEAC01000415.1	+1	31600-31758		A
SCL	*PaSCL*	AEAC01013335.1	+2	41306-41445	Helix 2: 769bp	A
			+3	42215-42233		
NSCL	*PaNSCL*	AEAC01011813.1	−1	35707-35685	Basic: 536bp	A
			−3	35148-34998		
Mnt	*PaMnt1*	AEAC01010687.1	+1	58000-58008	Helix 2: 2123bp	B
			+2	55730-55876		
Mnt	*PaMnt2*	AEAC01009610.1	+1	110044-110202		B
Max	*PaMax1*	AEAC01023724.1	+3	2178-2336		B
Max	*PaMax2*	AEAC01012273.1	+1	1648-1803		B
Myc	*PaDm*	AEAC01001866.1	−3	39047-38889		B
USF	*PaUSF1*	AEAC01020973.1	−2	90699-90538	Loop: 88bp	B
			−3	90449-90399		
USF	*PaUSF2*	AEAC01025009.1	−1	32215-32075	Loop: 101bp	B
			−3	31973-31923		
MITF	*PaMitf*	AEAC01022111.1	+2	23396-23417	Basic: 5715bp	B
			+2	29133-29208	Loop: 7943bp	
			+1	37152-37233		
AP4	*PaCrp1*	AEAC01009287.1	+3	29454-29564	locate on two separate contigs	B
		AEAC01009292.1	+2	42008-42052		
AP4	*PaCrp2*	AEAC01008415.1	+2	21659-21817		B
TF4	*PaBmx*	AEAC01010529.1	−1	64180-64010		B
MLX	*PaMLX*	AEAC01022037.1	−1	166446-166282		B
SREBP	*PaSREBP*	AEAC01008623.1	−2	34731-34589	Helix 2: 82bp	B
			−3	34506-34497		
SRC	*PaTai*	AEAC01016429.1	−3	7000-6993	Basic: 2799bp	B
			−3	4193-4040		
Clock	*PaClk1*	AEAC01024883.1	−3	2390-2386	Basic: 117bp	C
			−3	2268-2121		
Clock	*PaClk2*	AEAC01008322.1	+3	17766-17927		C
AHR	*PaDys1*	AEAC01000476.1	−3	12657-12496		C
AHR	*PaDys2*	AEAC01002106.1	−1	11804-11649		C
AHR	*PaSs*	AEAC01016399.1	+2	38537-38698		C
Sim	*PaSim*	AEAC01018973.1	+2	52010-52171		C
Trh	*PaTrh*	AEAC01014876.1	−3	10700-10539		C
HIF	*PaSima*	AEAC01025720.1	−1	31971-31810		C
ARNT	*PaTgo*	AEAC01022806.1	−1	23646-23485		C
Bmal	*PaCyc*	AEAC01003406.1	+3	16767-16771	Basic: 1814bp	C
			+2	18586-18742		
Emc	*PaEmc*	AEAC01012314.1	+1	2998-3096		D
Hey	*PaHey*	AEAC01012628.1	+1	61852-62019		E
Hey	*PaStich1*	AEAC01002103.1	+3	5010-5177		E
H/E(spl)	*PaH1*	AEAC01020843.1	+1	70384-70389	Basic: 406bp	E
					Loop: 272bp	
			+2	70796-70891		
			+1	71164-71235		
H/E(spl)	*PaH2*	AEAC01020833.1	−2	8228-8223	Basic: 301bp	E
			−3	7921-7826	Loop: 3123bp	
			−3	4702-4631		
H/E(spl)	*PaSide*	AEAC01019264.1	−1	1124-957	locate on two separate contigs	E
		AEAC01019265.1	−2	1044-1039		
H/E(spl)	*PaE(spl)1*	AEAC01003976.1	+1	4951-4956	Basic: 1338bp	E
			+1	6295-6384	Loop: 2233bp	
			+2	8618-8695		
H/E(spl)	*PaE(spl)2*	AEAC01003969.1	−2	11262-11257	Basic: 199bp	E
			−3	11057-10890		
H/E(spl)	*PaE(spl)3*	AEAC01003972.1	+2	36986-37159		E
COE	*Pakn(col)*	AEAC01019320.1	−3	1427-1427	Helix 1: 197bp	F
			−2	1229-1096		

Secondly, one bHLH member, *PaTwi2*, had bootstrap value of 43 in MP tree. Nevertheless, it formed monophyletic clade with the same DmbHLH counterpart in NJ and ML tree with bootstrap values of 63 and 73, respectively. Two members, *PaAse2 and PaCato*, formed monophyletic clade with bootstrap values of 55 and 61 in ML trees, but formed monophyletic clade with weak bootstrap values (33 to 48) in NJ and MP trees. Consequently, we allocated them to defined families of orthologs according to the one or two trees with bootstrap values of over 50.

Thirdly, one bHLH member, *PaHey*, formed monophyletic clade in NJ and MP trees with bootstrap values of 98 and 45, respectively, but did not form monophyletic group in ML tree. Another PabHLH member, *PaH2*, formed monophyletic clade with bootstrap value 47 in MP tree, but did not form monophyletic clade in NJ and ML trees (marked with n/m* or n/m in Table 1). Albeit with insufficient statistical support, we tentatively defined orthologs for them because they all have one or two bootstrap support to testify their orthology to the correspondent *D. melanogaster* ortholog. Obviously, these classifications can be regarded as arbitrary and ought to be modified upon new data available. From a certain perspective, this phylogenetic divergence of bHLH motif sequences between *H. saltator* and *D. melanogaster* probably implies that these two insect species have evolved in quite different circumstances.

Finally, the remaining 8 members named *PaAse1*, *PaMnt2*, *PaCrp2*, *PaClk2*, *PaDys2*, *PaE(spl)1*, *PaE(spl)2* and *PaE(spl)3* did not form monophyletic clade or did not have sufficient bootstrap support in forming monophyletic clade with any single *D. melanogaster* homologue in all three phylogenetic trees constructed. They were identified through constructing phylogenetic trees with AmbHLH family members accordingly. Six members, namely *PaAse1*, *PaMnt2*, *PaCrp2*, *PaClk2*, *PaE(spl)2* and *PaE(spl)3*, were identified with sufficient confidence for all the bootstrap values were over 50 in all the constructed trees. The rest two members, *PaDys2* and *PaE(spl)1*, formed monophyletic clade in NJ and MP trees with bootstrap values ranging from 50 to 98. We assigned orthologs for them according to the two trees with bootstrap values over 50, though they did not form monophyletic group in ML trees.

Through prediction by SMART using the full sequence of identified bHLH members whose protein accession number were available (Additional file 3), we found that: a) Among members of group C, there are 4 sequences having one bHLH, one PAC (Motif C-terminal to PAS motifs) [26] and two PAS (PER-ARNT-SIM homology) domains, while EFN88377.1 has one bHLH, and two PAS domains. The remaining one (EFN80844.1) only has bHLH domain. b) For group E, all of the PabHLHs have bHLH and Orange domains, one of which, however, has Orange with scores less significant than the required threshold. And all of them ended with “WRPW” peptide. c) The rest groups were predicted to only have bHLH domains. These results suggest that our analyses are consistent with the previous reports [3,27,28], and it is conceivable that these domains may cooperate and thereby confer particular functions on the proteins containing them [9].

### Protein sequences and genomic coding regions of *H. saltator* bHLH genes

Protein sequence accession numbers of the 57 identified PabHLH motifs were listed in Table 1. As we have seen, there are only 46 PabHLH motifs whose protein sequence accession numbers were found in *H. saltator* genome database (shown as ‘EFN’ plus number). Protein sequences of the other 11 PabHLHs, namely *PaAto*, *PaNet*, *PaSage*, *PaPxs*, *PaMnt2*, *PaCrp1*, *PaCrp2*, *PaClk1*, *PaDys1*, *PaDys2*, and *PaTgo*, were not found in current database. The coding regions, intron location and length of 57 PabHLH motifs are listed in Table 2. The intron analysis shows that 22 PabHLH members have introns in the coding regions of their bHLH motifs. It should be noted that: a) coding regions of 18 PabHLH motifs have one intron, among which those of 6 PabHLH motifs have introns in the basic region, 7 have introns in the helix 1 region, 2 have introns in the loop region, and 3 have introns in the helix 2 region. b) Coding regions of 4 PabHLH motifs have two introns, all of which are in the basic and loop regions. Thus, altogether, coding regions of these 22 PabHLH motifs have 26 introns. In addition, 2 PabHLHs named *PaCrp1* and *PaSide* locate on two separate contigs in the genome (Table 2). The longest intron in coding regions of PabHLH motifs is 7,943 bp (base pairs), the shortest one is only 82 bp, and the average length of introns is 1,391 bp. While in pea aphid, fruit fly and honey bee, there are 26, 18 and 9 bHLH members having introns in coding regions of their bHLH motifs, and the total number of introns identified is 34, 20 and 9 with the longest one of 30,718, 11,845 and 4,460 bp, the shortest one of 62, 57 and 72, and the average length of 4,193, 1,082 and 1,326 bp, respectively [14,16].

In summary, the number of PabHLHs having introns is more than many other insect species, and they are found to have introns with average length only inferior to those of pea aphid. Moreover, PabHLHs have the shortest length of intron only higher than those of fruit fly and honey bee. PabHLH genes are more intron-dense than those of many other insects, indicating that *H. saltator* either gained introns at a faster rate or lost introns at a slower rate than other insects [29]. Previously hypothesized mechanisms of intron gain mainly involve intron transposition [30], transposon insertion [31], tandem genomic duplications [32], intron transfer [33], insertion of a Group II intron [30], intron gain during double strand break repair [34] and intronization [35,36]. Three previously hypothesized mechanisms of intron loss include Reverse Transcriptase-Mediated Intron Loss (RTMIL) [37], Meiotic recombination [29] and genomic deletions [38]. Notably, the genome of *H. saltator* contains copies of specific transposable element (TE) families, which differs significantly with other species (i.e. *Camponotus floridanus* and *A. mellifera* have very few TEs) [22,39]. We infer that there may be some relationships between the formation of intron in PabHLHs and TEs. Nevertheless, whether the more intron-dense introns are due to growing faster or losing slower needs further investigations.

The existence of EST (expressed sequence tag) sequence corresponding to identified bHLH motifs is an indication of genuine bHLH gene at least at the transcriptional level. However, we were unable to find any EST sequences for our identified PabHLHs due to the unavailability of *H. saltator* EST database at time of our survey. Therefore, further verification of our identified PabHLH members awaits expansion of *H. saltator* nucleotide sequence data and experimental cloning of the genuine *PabHLH* genes. It would thus be of particular interest to study whether the bHLH family members identified in *H. saltator* are expressed during various developmental processes and how their expression patterns may be related to those of other insect counterparts.

### bHLH repertoire of the *H. saltator* and other insect species

The above searches and analyses enabled us to define orthologs of families for 57 PabHLHs. This figure is comparable with 50, 51, 52, 54 and 59 bHLH members in the red flour beetle, honey bee, domestic silkworm, pea aphid and fruit fly, respectively (Table 3). According to Table 3, all of these five insect species lack genes of families Oligo, MyoRb, and Figα, and many of the families have the same number of genes, such as E12/E47, Ngn, Beta3, Net, Paraxis, Hand, PTFa, SCL, NSCL, SRC, Myc, SREBP, ARNT, Trh, HIF, Emc and COE. The fact that comparable number of bHLH families and similar orthologs were detected strongly suggests that, despite our analysis was made on a draft version of the *H. saltator* genome sequence, the set of PabHLH we retrieved is likely to be almost complete, and hence gives a highly accurate view of the bHLH repertoire of a ponerine ant. The major obvious difference, other than total genes, is the discrepancy of H/E (spl) family members. *D. melanogaster* have 11 to 12 H/E(spl) genes while other insects have 5 to 6. *H. saltator* has fewer genes in families ASCa, and Clock than *D. melanogaster*. It is conspicuous that *H. saltator* has one more gene in families Twist, Mnt, Max, USF, AHR and AP4 than most other insect species previously reported. Thirteen PabHLH families have more than one member (accounted for about 29% of the families), while in most other insects, families with more than one member are fewer (mean value 16%; range 13% to 20%). This suggests that some of the *H. saltator* bHLH genes have been originated through duplications. Moreover, a feature to be noted is that neither of the two families, namely Delilah and MyoRb, was found. Whether *H. saltator* does have fewer members of these families, or if it was due to incompleteness of the genome sequences remains for further exploitation. Therefore, it can be thought that additional bHLH members may be found after a newer and higher quality version of *H. saltator* genome sequences is released.

**Table 3:** A comparison on bHLH family members from six insect species

**Group**	**Family name**	**P.a.**	**A.m.**	**B.m.**	**T.c.**	**D.m.**	**A.p.**
A	ASCa	2	2	4	3	4	0
A	ASCb	0	0	0	0	0	1
A	MyoD	1	1	1	1	1	0
A	E12/E47	1	1	1	1	1	1
A	Ngn	1	1	1	1	1	1
A	NeuroD	0	0	0	1	0	0
A	Atonal	3	3	1	3	3	3
A	Mist	2	2	1	1	1	2
A	Beta3	1	1	1	1	1	1
A	Oligo	0	0	0	0	0	0
A	Net	1	1	1	1	1	1
A	Delilah	0	0	1	2	1	1
A	Mesp	1	1	1	0	1	1
A	Twist	2	1	1	1	1	1
A	Paraxis	1	1	1	1	1	1
A	MyoRa	1	1	1	1	1	1
A	MyoRb	0	0	0	0	0	0
A	Hand	1	1	1	1	1	1
A	PTFa	1	1	1	1	1	1
A	PTFb	2	1	1	2	2	2
A	SCL	1	1	1	1	1	1
A	NSCL	1	1	1	1	1	1
B	SRC	1	1	1	1	1	1
B	Figα	0	0	0	0	0	0
B	Myc	1	1	1	1	1	1
B	Mad	0	0	0	1	0	1
B	Mnt	2	1	1	1	1	1
B	Max	2	1	1	1	1	3
B	USF	2	2	1	1	1	1
B	MITF	1	1	1	1	1	0
B	SREBP	1	1	1	1	1	1
B	AP4	2	1	1	1	1	1
B	MLX	1	1	1	0	1	1
B	TF4	1	1	1	1	1	2
C	Clock	2	2	3	2	3	2
C	ARNT	1	1	1	1	1	1
C	Bmal	1	1	2	1	1	1
C	AHR	3	2	3	1	2	2
C	Sim	1	1	1	0	1	1
C	Trh	1	1	1	1	1	1
C	HIF	1	1	1	1	1	1
D	Emc	1	1	1	1	1	1
E	Hey	2	2	2	1(2?)	1(2?)	3
E	H/E(spl)	6	6	5	5(6?)	11(12?)	6
F	COE	1	1	1	1	1	1
	Total number of bHLH members	57	51	52	50	59	54
	Number of bHlH families with two or more members	13	8	6	6	6	9

Data of *P.a.* (ponerine ant) were from this study. Those of *A.m.* (*Apis mellifera*) and *B.m.* (*Bombyx mori*) were from Wang et al. [2007, 2008]. Those of *T.c.* (*Tribolium castaneum*) and *D.m.* (*Drosophila melanogaster*) were from Simionato et al. [2007]. Those of *A.p.* (*Acyrthosiphon pisum*) were from Dang et al. [2011].

**Figure 4:**
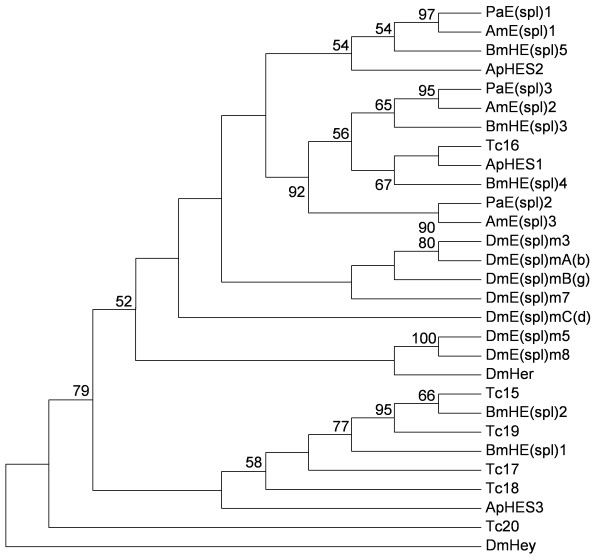
**Evolutionary relationships among E(spl) family members from six insect species.** This tree is based on a multiple alignment that includes all members of E(spl), which has been rooted using the closely related *Hey* gene from *D. melanogaster* as outgroup. And only bootstrap values more than 50 are shown.

We also executed several additional alignments consisting of only those bHLH sequences that belong to a particular family from 6 insect species above mentioned (Additional file 4). Based on these alignments, the in-family NJ trees were constructed (see Materials and methods) which has been rooted using a fruit fly bHLH sequence from the related family as outgroup. From our phylogenetic analyses (the trees not shown), 34 PabHLHs and AmbHLHs formed monophyletic clade with high bootstrap values (55 to 100), while the members of other insect species were fewer. So we concluded that the PabHLH genes, to a certain extent, have closer phylogenetic relationships with *A. mellifera* than with other insect species. Additionally, figure 4 shows a typical phylogenetic tree of a family containing the *E(spl)* family of the five insect species. The members of fruit fly *Esplm3*, *EsplmBg*, *EsplmAb* and *Esplm7* clustering together may reveal that these are the result of species-special duplication.

## Conclusions

By utilizing the 45 representative bHLH domains and 59 identified DmbHLHs as query sequences, 57 bHLHs encoded in *H. saltator* genome sequences were identified. It was necessary to use DmbHLH sequences as query motifs to detect six additional bHLHs found in *H. saltator*, namely *PaCato*, *PaAmos*, *PaCrp2*, *PaDys1*, *PaStich1* and *PaH2*, respectively. Since the 45 representative bHLH sequences were mainly from mouse [9,17], it was more reasonable that we assign relationships according to phylogenetic analysis with DmbHLH members. Additionally, for having no corresponding orthologous genes in *D. melanogaster*, orthology of 8 bHLHs, namely *PaAse1*, *PaMnt2*, *PaClk2PaDys2*, *PaE(spl)1*, *PaE(spl)2* and *PaE(spl)3*, were defined by in-group phylogenetic analyses with *A. mellifera* bHLHs. The in-family phylogenetic analyses suggest that the PabHLH genes have closer phylogenetic relationships with *A. mellifera* than others. Among all PabHLH members, protein sequences of 11 PabHLH motifs were not found in any protein databases. We must however caution that we might have missed some bHLHs and/or that we might have included bHLH domains from some pseudogenes. Nevertheless, these data will provide information for further research to obtain a qualitatively accurate assessment of bHLH complement from the information available.

## Methods

### *H. saltator* bHLH sequence search and primary selection

Candidate genomic sequences encoding bHLH motifs were identified using BLAST, NetGene2 and EditSeq program (version 5.01), prepared and improved with manual checking and phylogenetic analysis. As a starting point, both lists of 59 DmbHLH motifs and the 45 representative bHLH families were retrieved from the additional files of previous reports [17]. Each sequence was used as a query sequence to perform tblastn search against *H. saltator* genome sequence database ( http://www.ncbi.nlm.nih.gov/sutils/genom_table.cgi?organism=insects). The expect value (*E*) was set at 10 in order to detect all possible bHLH sequences. Redundant sequences were manually identified and subsequently discarded on purpose of keeping only one sequence with the same contig number, reading frame, and coding regions. Next, the sequences were examined again to check whether the obtained amino acids have covered the full bHLH motif or not. If not, the corresponding subject nucleotide sequence was retrieved and translated using EditSeq program (version 5.01) of the DNAStar package to add the missing amino acids on two ends of this motif. In case where a query sequence composed of two or three *H. saltator* coding regions, intron splice sites were assessed using the online program NetGene2 ( http://www.cbs.dtu.dk/services/NetGene2/) to find location of intron between the separate coding regions.

Moreover, in order to detect whether the retrieved bHLH sequence has corresponding protein sequences deposited in GenBank, each of the retrieved candidate bHLH sequences was used to make similarity searches using blastp algorithm against the NCBI nr database by limiting the query organism to *H. saltator*. And tblastn search was conducted against the NCBI Expressed Sequence Tag (EST) database to examine whether there were ESTs corresponding to the identified *H. saltator* bHLHs.

Finally, each obtained sequence was examined for their amino acid residues at the 19 conserved sites [40] by manual checking. Accordingly, a sequence with less than 9 mismatches could be a potential bHLH motif [41]. Therefore, sequences having no less than 10 conserved amino acid residues among the 19 conserved sites were regarded as potential PabHLH (ponerine ant bHLH) members, except for Emc and COE family members which have around 30 and 50 amino acids in their HLH motif respectively and thus the minimum conserved amino acids were adjusted to 5 and 8 respectively. Sequences failing to meet the above requirements were discarded.

### Multiple sequence alignments

To examine sequence features of these PabHLH domains, we performed multiple sequence alignment of all the potential bHLH sequences improved by the aforementioned approaches using Clustal W program (version 5.0) implemented in MEGA 5 [42] using the default settings. The aligned PabHLH motifs were highlighted in GeneDoc Multiple Sequence Alignment Editor and Shading Utility (Version 2.6.02) [43] and then copied to rich text file (RTF) for further annotation.

### Phylogenetic analysis

Three different algorithms were employed for phylogenetic reconstruction: distance neighbour-joining (NJ), maximum parsimony (MP), and maximum likelihood (ML). In general, the phylogenetic trees constructed by the different algorithms were congruent and displayed very similar topologies. First, distance trees were constructed with the neighbor-joining (NJ) algorithm [44] using PAUP 4.0 Beta 10 [45] relying on the step matrix constructed from Dayhoff PAM 250 distance matrix by R. K. Kuzoff ( http://paup.csit.fsu.edu/nfiles.html). Each PabHLH motif sequence was then used to conduct in-group phylogenetic analysis with DmbHLH motif sequences. The in-group phylogenetic trees were performed with NJ and MP algorithms implemented in PAUP program, as well as with ML algorithm using the program TreePuzzle 5.2 [46]. The NJ tree was bootstrapped with 1,000 replicates to provide information about their statistical reliability, while the MP analysis was generated with heuristic search of 100 bootstrap replicates. For ML reconstruction, the parameters were set as follows: quartet-puzzling tree-search procedure, 25,000 puzzling steps and the substitution model set to Jones-Taylor-Thornton [47]. Other parameters were of default values. Additionally, all members from each bHLH family of ponerine ant, red flour beetle, honey bee, domestic silkworm, pea aphid and fruit fly were used to perform phylogenetic analyses, which were termed as in-family phylogenetic analysis because the bHLH motifs for a particular analysis were from the same bHLH family. Each in-family phylogenetic tree was rooted using fruit fly bHLH sequence from a different but related family.

### Domain predicting

In order to further ascertain the reliability of the retrieved motifs and to examine whether the full-length protein sequences, especially those of Group C, E and F, contain additional characteristic domains, we carried out the predictions of protein domain architectures using Simple modular architecture research tool (SMART, http://smart.embl.de/) available online [48,49].

## Abbreviations

Pa: Ponerine ant, *Harpegnathos saltator*; bHLH: Basic helix-loop-helix; PabHLH: Ponerine ant bHLH; Am: *Apis mellifera*; Ap: *Acyrthosiphon pisum*; Bm: *Bombyx mori*; Dm: *Drosophila melanogaster*; Tc: *Tribolium castaneum*.

## Competing interests

The authors declare that they have no competing interests.

## Supplementary Material

Additional file 1 **The 45 families of representative bHLH motif sequences and 59*Drosophila melanogaster*bHLH (DmbHLH) motifs.** Click here for file

Additional file 2 **Amino acid sequences of 57 ponerine ant bHLH motifs.** The ponerine ant bHLH family members are arranged as those in **Tables** 1**and**2, in which their family assignment, protein and coding region information can be found accordingly. Click here for file

Additional file 3 The full sequences of identified PabHLH members whose protein accession numbers are available. Click here for file

Additional file 4 The bHLH sequences belonging to a particular family from 6 insect species, named ponerine ant, red flour beetle, honey bee, domestic silkworm, pea aphid and fruit fly. Click here for file
