# Supplementary material for: A genome-wide identification and analysis of the basic helix-loop-helix transcription factors in the ponerine ant, Harpegnathos saltator
Source: BMC Evol Biol. 2012 Aug 31;12:165. doi: 10.1186/1471-2148-12-165 (PMC3527142; doi:10.1186/1471-2148-12-165)
Supplement: Additional file 1 — The 45 families of representative bHLH motif sequences and 59Drosophila melanogasterbHLH (DmbHLH) motifs. [file 1471-2148-12-165-S1.doc]

**Representative bHLH motif sequences of 45 families**

>ASCa_MmMash1

AVARRNERERNRVKLVNLGFATLREHVPNGAANKKMSKVETLRSAVQYIRALQ

>ASCb_MmMash3

FIRKRNERERQRVKCVNEGYARLRRHLPEDYLEKRLSKVETLRAAIKYISYLQ

>MyoD_MmMyoD

RRKAATMRERRRLSKVNEAFETLKRCTSSNPNQRLPKVEILRNAIRYIEGLQ

>E12E47_MmE2a

RRMANNARERLRVRDINEAFKELGRMVQLHLKSDKPQTKLLILHQAVAVILEQQ

>Ngn_MmMath4A

RRLKANNRERNRMHNLNAALDALREVLPTFPEDAKLTKIETLRFAHNYIWALT

>NeuroD_MmMath3

RRVKANARERTRMHGLNDALDNLRRVMPCYSKTQKLSKIETLRLARNYIWALS

>Atonal_MmMath1

RRLAANARERRRMHGLNHAFDQLRNVIPSFNNDKKLSKYETLQMAQIYINALS

>Mist_MmMIST1

RRLESNERERQRMHKLNNAFQALREVIPHVRADKKLSKIETLTLAKNYIKSLT

>Best3_MmBeta3

LRLNINARERRRMHDLNDALDELRAVIPYAHSPSVRKLSKIATLLLAKNYILMQA

>Oligo_MmOlg2

LRLKINSRERKRMHDLNIAMDGLREVMPYAHGPSVRKLSKIATLLLARNYILMLT

>Net_MmMATH6

RRLLANARERTRVHTISAAFEALRKQVPCYSYGQKLSKLAILRIACNYILSLA

>Delilah_BfDelilah

RRKSANSRERDRMHQINYAFEALRCVVPKLPPSATQDASHAGKMTKITTLRLAMNYISALQ

>Mesp_MmMesp1

QRQSASEREKLRMRTLARALHELRRFLPPSVAPTGQNLTKIETLRLAIRYIGHLS

>Twist_MmTwist

QRVMANVRERQRTQSLNEAFAALRKIIPTLPSDKLSKIQTLKLAARYIDFLY

>Paraxis_MmParaxis

QRQAANARERDRTQSVNTAFTALRTLIPTEPVDRKLSKIETLRLASSYIAHLA

>MyoRa_HsMyoRa

QRNAANARERARMRVLSKAFSRLKTSLPWVPPDTKLSKLDTLRLASSYIAHLR

>MyoRb_HsMyoRb

PAAANAARERSRVQTLRHAFLELQRTLPSVPPDTKLSKLDVLLLATTYIAHLT

>Hand_MmdHand

RRGTANRKERRRTQSINSAFAELRECIPNVPADTKLSKIKTLRLATSYIAYLM

>PTFa_HsPTFa

LRQAANVRERRRMQSINDAFEGLRSHIPTLPYEKRLSKVDTLRLAIGYINFLS

>PTFb_HsPTFb

QRQAANIRERKRMFNLNEAFDQLRRKVPTFAYEKRLSRIETLRLAIVYISFMT

>SCL_MmLyl1

RRVFTNSRERWRQQHVNGAFAELRKLLPTHPPDRKLSKNEVLRLAMKYIGFLV

>NSCL_MmHen1

YRTAHATRERIRVEAFNLAFAELRKLLPTLPPDKKLSKIEILRLAICYISYLN

>SRC_MmSRC1

CDTLASSTEKRRREQENKYLEGLAELLSANISDIDSLSVKPDKCKILKKTVDQIQLMK

>FIGα_MmFIGα

RRRVANAKERERIKNLNRGFAKLKALVPFLPQSRKPSKVDILKGATEYIQILG

>Myc_MmNMyc

RRRNHNILERQRRNDLRSSFLTLRDHVPELVKNEKAAKVVILKKATEYVHALQ

>Mad_MmMad4

NRSSHNELEKHRRAKLRLYLEQLKQLGPLGPDSTRHTTLSLLKAKMHIKKLE

>Mnt_MmMNT

TREVHNKLEKNRRAHLKECFETLKRNIPNVDDKKTSNLSVLRTALRYIQSLK

>Max_MmMax

KRAHHNALERKRRDHIKDSFHSLRDSVPSLQGEKASRAQILDKATEYIQYMR

>USF_MmUSF1

RRAQHNEVERRRRDKINNWIVQLSKIIPDCHADNSKTGASKGGILSKACDYIRELR

>MITF_MmMITF

NHNLSKFVERRRRFNINDRIKELGTLIPKSNDPDMRWNKGTILKASVDYIRKLQ

>SREBP_MmSREBP1

KRTAHNAIEKRYRSSINDKIVELKDLVVGTEAKLNKSAVLRKAIDYIRFLQ

>AP4_MmAP4

RREIANSNERRRMQSINAGFQSLKTLIPHTDGEKLSKAAILQQTAEYIFSLE

>MLX_MmMlx

RRITHISAEQKRRFNIKLGFDTLHGLVSTLSAQPSLKVSKATTLQKTAEYILMLQ

>TF4_MmTF4

RRRAHTQAEQKRRDAIKRGYDDLQTIVPTCQQQDFSIGSQKLSKAIVLQKTIDYIQFLH

>Clock_MmNPAS2

KRASRNKSEKKRRDQFNVLIKELSSMLPGNTRKMDKTTVLEKVIGFLQKHN

>ARNT_MmARNT2

ARENHSEIERRRRNKMTAYITELSDMVPTCSALARKPDKLTILRMAVSHMKSLR

>Bmal_MmBmal1

AREAHSQIEKRRRDKMNSFIDELASLVPTCNAMSRKLDKLTVLRMAVQHMKTLR

>AHR_MmAHR

AEGIKSNPSKRHRDRLNTELDHLASLLPFSPDIISKLDKLSVLRLSVSYLRAKS

>Sim_MmSim1

MKEKSKNAARTRREKENSEFYELAKLLPLPSAITSQLDKASIIRLTTSYLKMRV

>Trh_MmNPAS3

MKEKSRDAARSRRGKENFEFYELAKLLPLPAAITSQLDKASIIRLTISYLKMRD

>HIF_MmEPAS1

MKEKSRDAARSRRSKETEVFYELAHELPLPHSVSSHLDKASIMRLAISFLRTHK

>Emc_MmId2

SKLKELVPSIPQNKKVTKMEILQHVIDYILDLQ

>Hey_MmHey1

RKRRRGIIEKRRRDRINNSLSELRRLVPSAFEKQGSAKLEKAEILQMTVDHLKMLH

>HEspl_MmHES1

RKSSKPIMEKRRRARINESLSQLKTLILDALKKDSSRHSKLEKADILEMTVKHLRNLQ

>COE_MmCoe1

GTPGRFIYTALNEPTIDYGFQRLQKVIPRHPGDPERLPKEVILKRAADLVEALY

**59 *Drosophila* *melanogaster* bHLH (DmbHLH) motifs**

>ac

SVIRRNARERNRVKQVNNGFSQLDLSNGRRGIGPGANKKLSKVSTLKMAVEYIRRLQ

>sc

SVQRRNARERNRVKQVNNSFARLRDLTKGGGRGPHKKISKVDTLRIAVEYIRSLQ

>lsc

SVARRNARERNRVKQVNNGFVNLRQHLPQTVVNSLSNGGRGSSKKLSKVDTLRIAVEYIRGLQ

>ase

AVARRNARERNRVKQVNNGFALLREKIPEEVSEAFEAQGAGRGASKKLSKVETLRMAVEYIRSLE

>da

RRQANNARERIRIRDINEALKELGRMCMTHLKSDKPQTKLGILNMAVEVIMTLE

>nau

RRKAATMRERRRLRKVNEAFEILKRRTSSNPNQRLPKVEILRNAIEYIESLE

>tap

RRMKANDRERNRMHNLNDALEKLRVTLPSLPEETKLTKIEILRFAHNYIFALE

>Mistr

RRLESNERERMRMHSLNDAFQSLREVIPHVEMERRLSKIETLTLAKNYIINLT

>Oli

VRLNINARERRRMHDLNDALDELRSVIPYAHSPSVRKLSKIATLLLAKNYILMQQ

>cato

RRQAANARERKRMNGLNAAFERLREVVPAPSIDQKLSKFETLQMAQSYILALC

>ato

RRLAANARERRRMQNLNQAFDRLRQYLPCLGNDRQLSKHETLQMAQTYISALG

>amos

RRLAANARERRRMNSLNDAFDKLRDVVPSLGHDRRLSKYETLQMAQAYIGDLV

>net

RRIEANARERTRVHTISAAYETLRQAVPAYASTQKLSKLSVLRVACSYILTLS

>MyoR

QRNAANARERMRMRVLSSAYGRLKTKLPNIPPDTKLSKLDTLRLATLYIKQLI

>del

RRKTANARERTRMREINTAFETLRHCVPEAIKGEDAANTNEKLTKITTLRLAMKYITMLT

>sage

YRRTACDRERTRMRDMNRAFDLLRSKLPISKPNGKKYSKIESLRIAINYINHLQ

>Pxs

PRQKINARERYRTFNVNSAYEALRNLIPTEPMNRKLSKIEIIRLASSYITHLS

>twi

QRVMANVRERQRTQSLNDAFKSLQQIIPTLPSDKLSKIQTLKLATRYIDFLC

>Fer1

QRQAANLRERRRMQSINEAFEGLRTHIPTLPYEKRLSKVDTLKLAISYITFLS

>Fer2

QRQAANVRERKRIQRINSAFDELRVHVPTFPYEKRLSKIDTLRLAIAYISLLR

>Fer3

QRRAANIRERRRMFNLNEAFDKLRRKVPTFAYEKRLSRIETLRLAITYIGFMA

>Hand

KRNTANKKERRRTQSINNAFSYLREKIPNVPTDTKLSKIKTLKLAILYINYLV

>SCL

RKVFTNTRERWRQQNVSGAFAELRKLVPTHPPDKKLSKNEILRSAIKYIKLLT

>NSCL

YRTAHATRERIRVEAFNVSFAELRKLLPTLPPDKKLSKIEILKLAICYIAYLN

>Mnt

TREVHNKLEKERRAQLKECYDLLKKVLPMGDEDRKKTSNLTILDTAHKYVNSLS

>Max

KRAHHNALERRRRDHIKESFTNLREAVPTLKGEKASRAQILKKTTECIQTMR

>dm

KRNQHNDMERQRRIGLKNLFEALKKQIPTIRDKERAPKVNILREAAKLCIQLT

>USF

RRATHNEVERRRRDKINSWIFKLKEMLPEASTSPSTSGSTKGGILIKACEYIKSMQ

>Mitf

KKDNHNMIERRRRFNINDRIKELGTLLPKGSDAFYEVVRDIRPNKGTILKSSVDYIKCLK

>crp

RREIANSNERRRMQSINAGFQSLRSLLPRHEGEKLSKAAILQQTFQYIVELE

>bmx

RREAHTQAEQKRRDAIKKGYDSLQELVPRCQPNDSSGYKLSKALILQKSIEYIGYLN

>Mlx

RRAGHIHAEQKRRYNIKNGFDTLHALIPQLQLNPNAKLSKAAMLQKGADHIKQLR

>SREBP

KRSAHNAIERRYRTSINDKINELKNLVVGEQAKLNKSAVLRKSIDKIRDLQ

>tai

SQINKCNNEKRRREAENGYIEQLSEILTLNKRGDMTSTKPDKAAILNQVVRTYREIC

>clk

KRKSRNLSEKKRRDQFNSLVNDLSALISTSSRKMDKSTVLKSTIAFLKNHN

>Rst1JH

GREARNLAEKQRRDKLNASIQELATMVPHAAESSRRLDKTAVLRFATHGLRLQY

>gce

GREARNRAEKNRRDKLNGSIQELSTMVPHVAESPRRVDKTAVLRFAAHALRLKH

>dys

ANKSTKGASKMRRDLINAEIANLRDLLPLPQSTRQRLSQLQLMALVCVYVRKAN

>ss

DGVTKSNPSKRHRERLNAELDLLASLLPFEQNILSKLDRLSILRLSVSYLRTKS

>sim

MKEKSKNAARTRREKENTEFCELAKLLPLPAAITSQLDKASVIRLTTSYLKMRQ

>trh

RKEKSRDAARSRRGKENYEFYELAKMLPLPAAITSQLDKASIIRLTISYLKLRD

>sima

RKEKSRDAARCRRSKETEIFMELSAALPLKTDDVNQLDKASVMRITIAFLKIRE

>tgo

SRENHCEIERRRRNKMTAYITELSDMVPTCSALARKPDKLTILRMAVAHMKALR

>cyc

RKQNHSEIEKRRRDKMNTYINELSSMIPMCFAMQRKLDKLTVLRMAVQHLRGIR

>emc

SKLKDLVPFMPKNRKLTKLEIIQHVIDYICDLQ

>Hey

RKKRRGVIEKKRRDRINSSLTELKRLVPSAYEKQGSAKLEKAEILQLTVEHLKSLQ

>Stich1

DPLSHRIIEKRRRDRMNSCLADLSRLIPPQYQRKGRGRIEKTEIIEMAIRHLKHLQ

>h

RRSNKPIMEKRRRARINNCLNELKTLILDATKKDPARHSKLEKADILEKTVKHLQELQ

>dpn

RKTNKPIMEKRRRARINHCLNELKSLILEAMKKDPARHTKLEKADILEMTVKHLQSVQ

>side

KRTNKPLMEKRRRARINQSLAILKALILESTKTQNAKNGEGQAKHTKLEKADILELTVRHFQRHR

>Esplm3

RKVMKPLLERKRRARINKCLDDLKDLMVECLQQEGEHVTRLEKADILELTVDHMRKLK

>Esplm5

LKVKKPLLERQRRARMNKCLDTLKTLVAEFQGDDAILRMDKAEMLEAALVFMRKQV

>Esplm8

QKVKKPMLERQRRARMNKCLDNLKTLVAELRGDDGILRMDKAEMLESAVIFMRQQK

>Esplm7

RKVMKPLLERKRRARINKCLDELKDLMAECVAQTGDAKFEKADILEVTVQHLRKLK

>EsplmBg

RKVMKPMLERKRRARINKCLDELKDLMVATLESEGEHVTRLEKADILELTVTHLQKMK

>EsplmCd

RKVTKPLLERKRRARMNLYLDELKDLIVDTMDAQGEQVSKLEKADILELTVNYLKAQQ

>EsplmAb

RKVMKPMLERKRRARINKCLDELKDIMVECLTQEGEHITRLEKADILELTVEHMKKLR

>Her

REVFKPMMERKRRSRINRCLDFIKDLLQEVSHLDGETMAKMDMGDVLELAVHHLSKKN

>Kncol

ALNEPTIDYGFQRLQKLIPRHPGDPEKLQKEIILKRAADLVEALY
