# Supplementary material for: A genome-wide identification and analysis of the basic helix-loop-helix transcription factors in the ponerine ant, Harpegnathos saltator
Source: BMC Evol Biol. 2012 Aug 31;12:165. doi: 10.1186/1471-2148-12-165 (PMC3527142; doi:10.1186/1471-2148-12-165)
Supplement: Additional file 2 — Amino acid sequences of 57 ponerine ant bHLH motifs. The ponerine ant bHLH family members are arranged as those in Tables 1and2, in which their family assignment, protein and coding region information can be found accordingly. [file 1471-2148-12-165-S2.doc]

>*PaAse1*

SVARRNARERNRVKQVNNGFATLRQHIPQSVAQSLGSNTAGTHGGSRAGSKKLSKVETLRMAVEYIRSLK

>*PaAse2*

AVARRNARERNRVKQVNNGFATLRQHIPSHIAAGYGDRGKKLSKVETLRMAVEYIRGLQ

>*PaDa*

RRQANNARERIRIRDINEALKELGRMCMTHLKTDKPQTKLGILNMAVEVIMTLE

>*PaNau*

RRKAATLRERRRLRKVNEAFEVLKRRTSNNPNQRLPKVEILRNAIEYIESLE

>*PaTap(bp)*

RRIKANDRERHRMHTLNDALERLRMALPTFPEDTKLTKIETLRFAHNYIWALS

>*PaMistr1*

RRLESNKRERMRMHSLNDAFQSLREVIPHVTKGRRLSKIETLTLAKNYIVALT

>*PaMistr2*

RRLESNERERMRMHSLNDAFEQLREVIPHVKMERKLSKIETLTLAKNYIMALT

>*PaOli*

VRLNINARERRRMHDLNDALDELRSVIPYAHSPSVRKLSKIATLLLAKNYILMQG

>*PaCato*

RRLAANARERRRMNGLNDAFDKLREVVPNLGTDHKLSKFETLQMAQSYIAALC

>*PaAto*

RRLAANARERRRMQNLNKAFDRLRTYLPSLGNDRQLSKYETLQMAQSYITALY

>*PaAmos*

RRLAANARERRRMNSLNDAFDRLRDVVPSLGNDRKLSKFETLQMAQTYIAALY

>*PaNet*

RRIEANARERTRVHTISAAFDTLRRAIPAYSHNQKLSKLSVLRIACSYIVTLT

>*PaMyoR*

PRNAANARERARMRVLSKAFCKLKTTLPWVPSDTKLSKLDTLRLAATYIAHLR

>*PaSage*

YKKSACDRERTRMRDMNRAFELLRSKLPICKPAGKKLSKIESLRHAITYIRHLQ

>*PaPxs*

QRYQANARERDRTHSVNTAFSALRTLIPTEPADRKLSKIETLRLASSYISHLD

>*Pa**Twi1*

QRVMANVRERQRTQSLNEAFAALRSVIPTLPSDKLSKIQTLKLATKYIEFLH

>*PaTwi2*

QRKMTNAKERQRTRDLNNAYDDLKKAIPFMSSEKMSKIQTLKLATKYILYLQ

>*PaFer1*

QRQAANMRERRRMQNINDAFEGLRAHIPTLPYEKRLSKVDTLKLAIGYINFLN

>*PaFer2*

QRHAANIRERKRMLSINSAFDELRVHVPTFPYEKRLSKIDTLRLAIAYIALLR

>*PaFer3*

QRRAANIRERRRMFNLNEAFDKLRRKVPTFAYEKRLSRIETLRLAITYIAFMG

>*PaHand*

RRNTANKKERRRTQSINNAFADLRDCIPNVPADTKLSKIKTLRLAASYIGYLM

>*PaSCL*

RKLFTNSRERWRQQNVSGAFAELRKLVPTHPPDKKLSKNEILRMAIKYISLLS

>*PaNSCL*

YRTAHATRERVRVEAFNLAFAELRKLLPTLPPDKKLSKIEILRLAICYIAYLN

>*PaMnt1*

TREVHNKLEKNRRAHLKECFELLKRMLPAQDEKKSSNLSILHAANQYIQTLT

>*PaMnt2*

KRNDSVSLYCCRRAHLRTCLEKLKLLVPLGPETSRHTTLGLLTKAKRFIKVNH

>*PaMax1*

KRAHHNALERKRRDHIKDSFSSLKNAVPTLQAEKAASRAQILKKAAEYIQTMR

>*PaMax2*

KREHHNYLERKRRDDLKMVFFHLKNNVPTILKGKASRAVILTKTIEYIQKMR

>*PaDm*

RRIQHNTMEKRRRVYMASLFQQLRSLIPHPNPNFKMPKVRILMEAANYCKNLH

>*PaUSF1*

RRATHNEVERRRRDKINNWIAKLGKIIPECNVTGTTTNSSSNSGGEGKANYETQSKGGILAKACEYIGELR

>*PaUSF2*

RRATHNEVERRRRDKINSWITKLGKLLPDCDQNTNGEGDAKVNFESQSKGGILARACEYITKLK

>*PaMitf*

KKDNHNMIERRRRFNINDRIKELGTLLPKTNDPYYEIVRDVRPNKGTILKSSVEYIKLLK

>*PaCrp1*

RREIANSNERRRMQSINAGFQSLRSLLPHHEGEKLSKAAILQQTAEYIYQLE

>*PaCrp2*

LYSKAKTWERDRRKRMNAYFKTLADLLPPHQEGRKRNKVDILIHASNYIKDLH

>*PaBmx*

RREAHTQAEQKRRDAIKKGYDSLQDLVPTCQHTDSSGYKISKATVLQKSIDYIQFLL

>*PaMLX*

RRVGHIHAEQKRRYNIKNGFDMLHSLIPQLNQNPNTKLSKAAMLQKGADYIRQLR

>*PaSREBP*

KRSAHNAIERRYRTSINDKIIELKNIIVGVEAKLNKSAILRKTIDYIRFLQ

>*PaTai*

SQMNKCLNEKRRRTQENLYIDELAELISSTDMSSGKTDKCQILQRTVDQVRRQI

>*PaClk1*

KRKSRNLSEKKRRDQFNMLVNELGSMVSANTRKMDKSTVLKSTILFLKNHN

>*PaClk2*

PRASRNMAEKQRRDNLNTNISTMAALLPIVAGSSRRMDKISILRLAAAFLRTQY

>*PaDys1*

ASKSTKGASKLRRDLINAEIANLRDLLPLPPSTRQRLSQLQLMALVCVFLRKAN

>*PaDys2*

KKREHALALAPRIHGKNNARSGLRHTPFSPLSLRLSPAQTLEDAIAFRGARS

>*PaSs*

DGVTKSNPSKRHRERLNAELDTLASLLPFEQNILSKLDRLSILRLSVSYLRTKS

>*PaSim*

MKEKSKNAARSRRVKENQEFLELAKLLPLPAAITTQLDKASIIRLTTSYLKMRA

>*PaTrh*

RKEKSRDAARSRRGKENFEFYELAKMLPLPAAITSQLDKASIIRLTISYLKLRD

>*PaSima*

RKERSRDAARYRRSRETDIFADLAAVLPVAPQQAAHLDKASVMRLAIAYLKVRA

>*PaTgo*

CRENHCEIERRRRNKMTAYITELSDMVPTCSALARKPDKLTILRMAVAHMKALR

>*PaCyc*

KKQNHSEIEKRRRDKMNTYITELSAMVPMCHAMSRKLDKLTVLRMAVQHLKTIL

>*PaEmc*

TKLRSLVPDMPRKRKLSKLEVIQRVIEYICDLQ

>*PaHey*

RKRRRGMIEKKRRDRINASLGELRRLVPAAARDPHSGKLEKAEILQLTVEHLRTLR

>*PaStich1*

DPMSHRIIEKRRRDRMNNCLADLSRLIPAEYLKKGRGRVEKTEIIEMAIRHMKHLQ

>*PaH1*

RRSNKPIMEKRRRARINNSLNDLKTLVLDAMKKDPSRHSKLEKADILEMAVKHMENLQ

>*PaH2*

RKSNKPIMEKRRRARINQSLDELKALVLDAMKKDPTRHSKLEKADILEMAVKHIQTVH

>*PaSide*

RRANKPLMEKRRRARINQSLAALKALILDSARLENTKHSKLEKADILELTVRHLQRQR

>*PaE(spl)1*

CKITKPLLERKRRARINRCLDELKNIMVDALETERENISKLEKADILELTVRHLQRLQ

>*PaE(spl)2*

RKVMKPMLERKRRARINRCLDELKELMVTALAGDGENVAKLEKADILELTVRHLHKLQ

>*PaE(spl)3*

RKVMKPMLERKRRARINRCLDELKDLMVTALQAEGENVAKLEKADILELTVRHLHTLR

>*Pakn(col)*

SLNEPTIDYGFQRLQKLIPRHPGDPEKLPKEIILKRAADLAEALY
