# Supplementary material for: A genome-wide identification and analysis of the basic helix-loop-helix transcription factors in the ponerine ant, Harpegnathos saltator
Source: BMC Evol Biol. 2012 Aug 31;12:165. doi: 10.1186/1471-2148-12-165 (PMC3527142; doi:10.1186/1471-2148-12-165)
Supplement: Additional file 3 — The full sequences of identified PabHLH members whose protein accession numbers are available. [file 1471-2148-12-165-S3.doc]

>gi|307207747|gb|EFN85365.1| Achaete-scute complex protein T8 [Harpegnathos saltator]

QTLMGLKEESVLPIMLSVQQQHHHHGLHGTSSAQTHRGNVIVSTNSMPANNNNNSGTMQHGCKRSKIYGQPTGPYSTVPHQPASVARRNARERNRVKQVNNGFATLRQHIPQSVAQSLGSNTAGTHGGSRAGSKKLSKVETLRMAVEYIRSLKRLLEEHDGNNESSGSSPTSSTSSNSPMCGSENGVGSDSGHHPAELRLRGSVTGEVHHHDSPHGSDLHRHQHLRQNPSPTFVPTTCSEASSSPTPSFVSETSSAGSQGYSTSGTLYAMHSDSYDNYEPMSPEDEVLLDAITWWQQSQ

>gi|307207748|gb|EFN85366.1| Achaete-scute-like protein 1a [Harpegnathos saltator]

MATMGVTVFCNRVQINGNDQEQLMLLRTLQDNESNIISNQPLIVPKSNTASNTTSSTAVLPPYDPSRLKKHGKNGQPPPVAVARRNARERNRVKQVNNGFATLRQHIPSHIAAGYGDRGKKLSKVETLRMAVEYIRGLQRLLAEADGVEYDSSTGAGAQCVPSPTSSVVSSTHNGSGERLALEDDVLAAEDDEGEQLDEEEDGSNVKSRITLSRLSPNRTAEPSPREYYASPGGDEENLEPRTVTSGQNFSRNLSPNSAASPPSEYYTQNEENLEPQILSPYSGAGDSEEGATTIYATSPDTFSGALYYKQEITETGEFMDVVSWWEQEQGRLVHQQHTQRLTHV

>gi|307202902|gb|EFN82122.1| Protein daughterless [Harpegnathos saltator]

MSTFRGSAAVAANSGTGAPSPASLQYSHSPGAPTTAPSASNAAPTTNQQTTPGDTLGKTLASVSTRIYPTDQSVSSYSSNPSTPVSSPPPLTGSAPGWAPGAAPVSPHFTADPNRGIHMGTRMEERLDDAINVLRNHAESQLGLHLGPVGPHGAIYSHTSPPQLDHLTSPHSAVTVTQPQGSYPGLTPTPDTDGSIKIERLPVTNAKKRKDPPDSSGGETKPSSSELAAAGVIGAATVNSTSQGKGTKRSRRYAEDEDDDPDMKAQREKERRQANNARERIRIRDINEALKELGRMCMTHLKTDKPQTKLGILNMAVEVIMTLEQQVRERNLNPKAACLKRREEEKAEDGPKLPGHLAAHIAHPHSHAHAHSQAPFPALPGPQPSLQHNPPQPQ

>gi|307198255|gb|EFN79255.1| Transcription factor SUM-1 [Harpegnathos saltator]

MMTLVTDAVPYGYETVTRPPYDGYRHHHLHHHHHHHHHRYRRVYTPTEPEYLQSYVSRRDTDDRARIVGGNNALTAAVASPLNYRDRSDRIRSQSRYTLDSDDASSVISADASADLESGSADADETNETNETGETNETSEANDGESIEHVPHPHVLDAGSPHGPRRCLLWACKACKKKTVTVDRRKAATLRERRRLRKVNEAFEVLKRRTSNNPNQRLPKVEILRNAIEYIESLEALLQGNRPSGHQDHPSTENMARIYNTHTHRVRDNCN

>gi|307191639|gb|EFN75119.1| Basic helix-loop-helix neural transcription factor TAP [Harpegnathos saltator]

MSSEYSFCSEGGFDELSSSSDSGFDVSFESPKHEVPTDTLFAPVKEEKRKKTRNTRCKSPAQVLRLKRNRRIKANDRERHRMHTLNDALERLRMALPTFPEDTKLTKIETLRFAHNYIWALSQTLGNADSGEITVNVGNVTVNIGENGNMITSSTGSCAVAAQKRLGPTHTGFPYPQERFVPEWQEYDCYSDQSVSPPMQYQPCYDQRMYHPHRPQHQLPPPPHHHMVHHTNMYQCL

>gi|307212534|gb|EFN88257.1| Class B basic helix-loop-helix protein 8 [Harpegnathos saltator]

MRSKVTSENTWETKWATESSQSGKGVRHAAKRTGTSGLRLSRSARGNTARERSLRRLESNKRERMRMHSLNDAFQSLREVIPHVTKGRRLSKIETLTLAKNYIVALTDVICAIRSEEQPEDQQTSVHEAQASSDGERLEPSVVRVNIASRSCGSETQNFYQSDHSRWEDDLNNCTGL

>gi|307192581|gb|EFN75769.1| Class B basic helix-loop-helix protein 8 [Harpegnathos saltator]

MRKLDALSGSDQDCGDREMYIDLDDASVDSLTGKRGRHHVEVAEESDSSGSERSPKQAKRRNRGGAPPTTRRRKSGISARERNLRRLESNERERMRMHSLNDAFEQLREVIPHVKMERKLSKIETLTLAKNYIMALTNVICEMRGEERPYTFVDGECGSSSGDSTGQLELSGGEQPDESSPTSIHETNNNSLLHEDLERRI

>gi|307192831|gb|EFN75891.1| Class B basic helix-loop-helix protein 5 [Harpegnathos saltator]

MRSYDGESSSTEDDDRREGLPEAHLQQGSWQRSASHPTWAWEHRNAVSNPENNCNLHPLPPQSAASLESVYSTPGASHPGVSAPPAAYSSEHTQSAPGRRTPLGAVGLGGFYFQQQPQPHASSSALSDENRPDERPGASRLNCPPKSKTTRQGKSVRLNINARERRRMHDLNDALDELRSVIPYAHSPSVRKLSKIATLLLAKNYILMQGNALEELRRVIAVLQSPHAHTTALPPTPVSYDLLQGFPGKLFQGVQEMQGLPTGDPQIVTAGGPPTDGTVASGESN

>gi|307202800|gb|EFN82083.1| Basic helix-loop-helix transcription factor amos [Harpegnathos saltator]

MSTTPPVSPRKGRRRSRDVPPSPTVLKRRRLAANARERRRMNGLNDAFDKLREVVPNLGTDHKLSKFETLQMAQSYIAALCDLLQRHDGKR

>gi|307202799|gb|EFN82082.1| Basic helix-loop-helix transcription factor amos [Harpegnathos saltator]

MTSYATFQQYDLLDSEGYGSASSPESNPRSNWHHQEIYPQPPRSRGSSCGSVSSVDSHQQQEYQEIGGAPNGSLHVTAGFNFADFYEGYYQEGCRGNEHQPLNGNGAKSVKDHHSKVIFRMNDQCVNYGDVPSRMGFNEGAASKQDPTGYAPKMEHHHPVGVFANSRADYTQSQEAVFATKSYAKNSGFMSRNEGHPYAQRTDVLYLGATYTVQRNDVGLTTNGQQIKCDQSRYHQRNDGLHVLPADYAGQKSKDAMQKMKNSAPGIEVLRKRRLAANARERRRMNSLNDAFDRLRDVVPSLGNDRKLSKFETLQMAQTYIAALYELLQRE

>gi|307196706|gb|EFN78165.1| Transcription factor 21 [Harpegnathos saltator]

MPRKRRASTSSLEEYPSEEKYAKDDMDSRAPRNAANARERARMRVLSKAFCKLKTTLPWVPSDTKLSKLDTLRLAATYIAHLRAVLKDDGEAHPESTKSLSLTLSWPFAIQNSNAAAMMNNSCNVSSSTSTGCNQYRGQQATHDIQNFHHSGHQGASTRHNQEQQLSYF

>gi|307196254|gb|EFN77900.1| Twist-related protein 1 [Harpegnathos saltator]

MKLKVRRKSSASMEEIQNQRVMANVRERQRTQSLNEAFAALRSVIPTLPSDKLSKIQTLKLATKYIEFLHQVLRSDLEKDDGSENIAGRSAKNAIIAARQSRDLPCSYMAHERLSYAFSVWRMEGDWNSNT

>gi|307196255|gb|EFN77901.1| Protein twist [Harpegnathos saltator]

MEDHIHTERSIFANVASDHKVYVPFMQQSQNVKPCVEGSSVPVLTPVGNTQTAQPQDYINYNIMSNDDVSAYPTNTSPANYNSNVDVINATGMRNIKYMNNCMSNYELQYLHDRYSAAACNHSSKCSGKHSEKKYANSSYYHMENVKCKDGEAISQSCDGHKSMEIDEPMVRPCSSNNYVMNFSLSSESEEKLVSIDNSDSQSEIVSSETSSCYKQCMDDDPENQRKMTNAKERQRTRDLNNAYDDLKKAIPFMSSEKMSKIQTLKLATKYILYLQFKNRCDFCQKISKFLLLHPYICFHISFSFYLLEYSNSFLAIFYFSKLS

>gi|307191968|gb|EFN75358.1| Pancreas transcription factor 1 subunit alpha [Harpegnathos saltator]

MYSMDNLERDMMNRQYMYEAHSFLGNPAIPALPPHCGVPTTATLPAIPSGSTGSTSGSEHYLYDENSSDNESVYSSDQENHARERSRSNRRSGASGKCPRQVQVQQRQAANMRERRRMQNINDAFEGLRAHIPTLPYEKRLSKVDTLKLAIGYINFLNELVRADKGNDPLTGNSGLSRCSSRDDSKKVIVRGEANFNYSPTGSGGNPFISHSLSWSRKSDISPNGMMYAKVWTPEDPRTSKNGTTFE

>gi|307200357|gb|EFN80609.1| Fer3-like protein [Harpegnathos saltator]

MANHHPAYDFEDRGQRIRHRVGTASRPADSTVACTSTQYYVGPSSDISEEDLAELPSCVYAGRSTQHVAHTSPHTHSPLHYHMPVSTPDHNDTEINGVEEGYYPNGSPYRIQRHAANIRERKRMLSINSAFDELRVHVPTFPYEKRLSKIDTLRLAIAYIALLREVLAARLDPLTYVERCLRGEINGERAEWNTSGSYSLIILESKLFPSK

>gi|307195685|gb|EFN77527.1| Protein Fer3 [Harpegnathos saltator]

MSYTEPLWEINGNQAPVIAAEMSQYVGGEIGSYPMWDGSVLYQAQPPTHPHVNEHGLYRQPCALLHQSRYTPNGRSPNLPSSTTKKPRRRVATVSQRRAANIRERRRMFNLNEAFDKLRRKVPTFAYEKRLSRIETLRLAITYIAFMGELLGIEPGSPKQEYIPREYYLPN

>gi|307215253|gb|EFN90007.1| Heart- and neural crest derivatives-expressed protein 2 [Harpegnathos saltator]

MMLGGYEAQAEYYPQWHQPYNYVTQLPYGPLGSVVTSAPDADSQSTYWGADSGISGDSSGAGSPTASTPPLIEEIGIQEPSYSPVQQYAQPSAGQHYAATLQYPQQQETSPHHHHHHLHHHHHHHHQSSHRRGEHEYANVQVGGQQQVIGGVVASLQQHLHQGVREGDVVPRPKRRNTANKKERRRTQSINNAFADLRDCIPNVPADTKLSKIKTLRLAASYIGYLMAVLESDEGEEPQTFRAEILSNGRRNKAAQPNQNESCLQSASNLGHEESTKSKGRTGWPQHMWALELKQESPTSQMQ

>gi|307203597|gb|EFN82626.1| T-cell acute lymphocytic leukemia protein 1 [Harpegnathos saltator]

MLTYSGCSGGTADTENGSPAESLLSGEGELPEEAGDSPGTSRDTEEEATLSDEFYSHDTDEEDDQSKRRRDRPSSLDGISPSGSGHLGSPPPRIGNMGVRKLFTNSRERWRQQNVSGAFAELRKLVPTHPPDKKLSKNEILRMAIKYISLLSSVLEWQKGQDRNGVQHQDVRIKCESHLSGQNPATYHTKSTVAYLKQEKQHPDTSHVFRINYGAQRHGSQHATCEKNGSNLLMIAPAGLNVSAAGKRCVTPVVVSQGSFHSNSNGNLIRSPASGRTSGFPKSPQVSSGTSMAPNGSSSSLTNNAPVTNGVGSSGSNCGQKRFKIEREEEEQGTSRDCRAPPPPAHSVPVRKRIKVTFVKDSVSGFRGDLCGVDRK

>gi|307205014|gb|EFN83537.1| Helix-loop-helix protein 1 [Harpegnathos saltator]

MKSFNCDFMSEDRAVSMANGGHGVVVNGVGAPVSGTLSREERRRRRRATQKYRTAHATRERVRVEAFNLAFAELRKLLPTLPPDKKLSKIEILRLAICYIAYLNHVLQA

>gi|307206058|gb|EFN84151.1| Max-binding protein MNT [Harpegnathos saltator]

MEHSSVHVRPRGGGKGALSTWCCWSISLPSKITGDHRKAGSPMPRSQNDHSAVREHRESAQQRQHQQQQHHRSVPSTCSATEQHTPSGMSLETLLQAAYYVEQEEKKRERLASTSSSSSSSEQHLFVSAAPLLSNHTYASTTRPRGVKIKKERPDSDDLFCEENMLIIDGEYSPHTIIDPNCFLWFSTNIDVMSIVCVCTAKVTEDTTSERKPSGSLGTRALRLGQEFSEKMGVLLLPDGTPQSHISLSAGIDVGVVGEANNNVSDLTDANSLSSQVTQPSTPTPTRVVGIINIVNNEEPPPPPPPPPPPPRRSSEWMPPPPKKKWIRHYLLEEDPLDISNQSNQSSQSIASRGSAVISSRGTPTASTNSSPCVIRSGTREVHNKLEKNRRAHLKECFELLKRMLPAQDEKKSSNLSILHAANQYIQTLTKKDLDYQRELQRLAEEKIAADQRLDMLKQEVVATWEHIDFSHIDALLARSATGADIMVVRNDTAEVEITGLPQGGTRYSSTSSLNSVTASSPQALQSPNATSNIHNQAATASIVCQTQDLNLARGSRESPPASSNSASSTPSISTPTQEKVATSPTANIVQQPHQLHLPISAQMLNTSQGLATIVPTLQHIGPGLRVIPGDTRQLLVTHTAGNNETRPLTLAVQNSSDQSRPLIAVQSNTGNEPRPVALVVHSSTASDNRVTFVHSNLSNNDRPLALAVQSSANDVRPVTFVHSGNEARPLVLATHSPALNVSNAQTRIRTSDSQTTHKMVGGVTLVGGNGSELARLPGGAELNILPANGLTLSHAGVSLQTAAAKPSSTVMQNVPSTESIAHIVGQHTPLSGLTPIVTPMTVVSQGNQVTAHILAPSSLAGKMITTPILKTVGQMPLVNAQYLNTTTLVKPVVVVSSPSTSTPPVSTTASSNTQPPSTTHSTV

>gi|307194222|gb|EFN76634.1| Protein max [Harpegnathos saltator]

MSDDDRDIDIESDDGDDSDSRLRHSNNTQYCSQAEKRAHHNALERKRRDHIKDSFSSLKNAVPTLQAEKAASRAQILKKAAEYIQTMRKKNVSQQQSIEDLKKQNTYLDAQIRLLEKARASGNFPPESCEVNTSEAMGLGNYNDTESESSDSETETAVRQPKKLKVAGLH

>gi|307204565|gb|EFN83237.1| Protein max [Harpegnathos saltator]

NIVNLFVQTEKREHHNYLERKRRDDLKMVFFHLKNNVPTILKGKASRAVILTKTIEYIQKMREETAVHQRITQLVGENMLLNDRSK

>gi|307213944|gb|EFN89178.1| hypothetical protein EAI_10151 [Harpegnathos saltator]

DEEIDVVSLASQRAKLAAQNKATPFRRPRGRPPLNRKRALPQEPEDHQPAPKRRQTRKLDAPGTSSSTDEEAQQDEQVKNKGRKKGKQKNKEKNPDRRIQHNTMEKRRRVYMASLFQQLRSLIPHPNPNFKMPKVRILMEAANYCKNLHEGAKTLSKLREDVTKKTAIVKQLECKLQRR

>gi|307196687|gb|EFN78146.1| Upstream stimulatory factor 2 [Harpegnathos saltator]

MDILGQHIEPQDASAVKDDSGTGIVIEEAEFVDCEGEPVGEDDMRYQEALAYRVVQVNSTAAIQSANEIELPVSQAGSNTVQVLTSPLNGQFYIVGNANDVFTAAQTSRSLVPRAATLQIETSRNCTTGLKKRDDRRRATHNEVERRRRDKINNWIAKLGKIIPECNVTGTTTNSSSNSGGEGKANYETQSKGGILAKACEYIGELRATNQNLSQCLRDNEKLRQEITALKQLITQLKHENHQLRSQITTSTGNTVEVVHLSP

>gi|307193180|gb|EFN76085.1| Upstream stimulatory factor 1 [Harpegnathos saltator]

MEVINRFDTMDESADENIMSDEAVGVVLEEAEIVDCDAEAEVDDDNVQYHLYAVNRSDNTITYKVMHVSDAHRENNEVSIATPVNNAVQVLASPLNGQFYVLSNGNDVVTSETARTVAPRVAKLQIEGSQNIITGLKKRDERRRATHNEVERRRRDKINSWITKLGKLLPDCDQNTNGEGDAKVNFESQSKGGILARACEYITKLKEDQEKLTQSLEENAQLTEEAKNLRQVVNDLKSENTKFKTQLLKDGAFILGP

>gi|307195724|gb|EFN77564.1| Microphthalmia-associated transcription factor [Harpegnathos saltator]

ELQIKPEPLLLTEAEIHALAKDRQKKDNHNMIERRRRFNINDRIKELGTLLPKTNDPYYEIVRDVRPNKGTILKSSVEYIKLLKNELTRMKQNELRHKQLEHQNRRLLLRVQELELQAKAHGLPVSDFNWASTSVTMLNSFPRSKLEQRKVHMPDLVTEEATSLSMSQFEDLMEDDTGGPVHGGDPMLSSPHLPPLSPPAAACHHGLADEDTLGSLAVTTSNSSSDMDIVA

>gi|307206343|gb|EFN84400.1| Max-like protein X [Harpegnathos saltator]

MADSLHKDGYEASGLMMHTGSGNIGSDSDMKLEPSSPTEKYTFSRCGSTGSVNTPSSSAHNTEDEDSDNKNSTISYKERRREAHTQAEQKRRDAIKKGYDSLQDLVPTCQHTDSSGYKISKATVLQKSIDYIQFLLQQKKRQEDERKALRKEVVGLRIMLANYEQIVKAHQTQPGHAEMRISDEMKFQVFQAIMERLFQSFNNISVANFGELTAYVFSWLEEHCKPQTLRDIVISVLQQLNISQIS

>gi|307195801|gb|EFN77615.1| MLX-interacting protein [Harpegnathos saltator]

MTSSSSQSGFHRAGNGNRESSSRDSRETIHSGHFMVSDFEAEAQDDEDELAVPVPDEEVTARLSIIAQPGFTVERFVANSSNDKSSLSIETSLNKLFQCMSLAYRQKLTSPKWNRFKGIRLRWKDKIRLNNVIWRCWHMQFILKQSTLVCQFASPLDVDTHNKPEAVVLEGKYWKRKLAAVTAEYKKWRMFYRNKILGWTNKDGTEMMESMDMLDWDSGSITTENYGMGIGSASGSISGAPGGESMMVDEDYMELMTDTLFSTISSNQPIYFPDPREIARGASLADFIQPSLGPLQPNLDDFMDTLEPLQEFLNSKLPPVPEEDDMFRNNSLNANYSDLDLMTPITQMNEINEDPTIKSEQSPQQQQQQQQTLAQEVQDANMQNLHYTAKIYTQPQPESSSVYRNSIAMTENYNAIQQNFETSVTSQSTREKNRSSSNSSSSRATSSRSSRVIQQAQQQSTYQATSGQQTQNSGFQPSPAQSSYAMEIQLTPVQNQQPVQIAALNDQSVNQISSIVAAAAAASSHVSNLVAVQHNFTQAANSPLQTQQRAIRPLPPTPPMSTKPYKIVQPQQCYKFSNLTQNFNAQQCKFNVNNFKTHPTQQVISVSAEQPSQSQILLQCRPSGLDLTAPALHPTKLLPQPTISSVETEEIFAVPKYQIKGRNRSRSSSSLTAPRIHPPLVSAVSDPALNLNNNVLLAHLLTNTSSTQVTTTQHITTPVTVQTMQTSNIQQAQPQQQVMQSSTCNQQILLNTNSQTHQSQNSPSSPKDSSNAHSPQTLSLSPLHSPMNIGSPLSPNRGYIKGESERGQYKEQRRVGHIHAEQKRRYNIKNGFDMLHSLIPQLNQNPNTKLSKAAMLQKGADYIRQLRAERNQMKEEMDNLKQQIECLNTSISNCQSMLPATGAPVSSHRTSKMKEMFNEYVRKRTRENWKFWIFSTLLEPLMLSFDASVSTASVNDLETSTILWVEQQCSLTRLRPAVLDCLRHLSTQTDILSNPARLPEEALEAINQTERRSSTQ

>gi|307207933|gb|EFN85492.1| Sterol regulatory element-binding protein 1 [Harpegnathos saltator]

MADPGGGWMPSQDNDFTNFQSNDSFNLNEVTGIDDLLTNCENELLKNENLFSDEALLSELDESIPMGGDTFDFLTLNNTDEFKDFKDFKDSSILEPPNSNVIITQSVTDNSQPIPCVQQDITSVQNRPQRISVATTPTVFNSQYTIPSNVSFNVQSPVVTLAPVPQQRQLILPAKIIKSESLVYSRGTQAVTSTSVPHQIRTLVNTANGTVLATGIPVVLDTEKVQINRLNTGTHVGVPRVREVKRSAHNAIERRYRTSINDKIIELKNIIVGVEAKLNKSAILRKTIDYIRFLQNSNTKLKAENMSLKMAAQRQNLRDLLTCGELTPPRSDSSEPSLSPAPAPLSPPSPSSVKDDTDALQNLHPMAASSNGGMRDHTRLTLCGFMLLFLAFNPLGILINNVGKFNYDYLNTKLDGRTILNYQADQSESDKLLWSSVILWLTNLILLAGGLCKLLRYGDPILPTDSKVFLELRRWRRQAEFNISKHEYNQAYRDLHQCLQYFGRSFPLSRTETFLATMWQIVRQVLHKLWLGRWVMQINKWLSDKSERQEAEMSAMEIAIVYQYTLCLRLSEGTRSPNLYFALCALNYAEAAGESMPKPLLAEIYVNTALCLKQSFFPYVHKYYLGKARVLLSSCTVPPKLKWIMTDEGARFLASYKWQYKNQRSDSEFTSQSSKADPVSYVARAYRDHLFEQCLRLLTGTAGEYHASSVLELGQIIMASAGVDASFPSTDQVSVATCEDEVGLWWGAVMCVAASWRLGEENSEAWSVVERKFPYKRNFQIGDSNSRSSPLPHAVLNVLQAAKHSSKLASMRFIDQAGLLLEQSMVYYHCKEQSSQQILLIQLLICDWLLELRTTLWQDLDAELDRSAANVSLANFQRDLACLRQLCQHIPSVLARVFLYEATARIMAGATPVKTQILLDRSSHHRNSRSSIICGKDRSQEQSNGEREHAVALYLTCRYLPTLLLVSPGARDGMLTEAAKTLERIGDRKRLLECYKLMQQLSSTITVN

>gi|307200819|gb|EFN80872.1| Nuclear receptor coactivator 1 [Harpegnathos saltator]

MSAITGSITKKRKKSDAKPQSQINKCLNEKRRRTQENLYIDELAELISSTDMSSGKTDKCQILQRTVDQVRRQIFPPSPLCMSMIDNIAVMPENMPAIFVIFFSPGDKAFVSVCA

>gi|307193316|gb|EFN76178.1| Circadian locomoter output cycles protein kaput [Harpegnathos saltator]

MDDDIDDKDDTKRKSRNLSEKKRRDQFNMLVNELGSMVSANTRKMDKSTVLKSTILFLKNHNEIAVRSRVHEIQEDWKPSFLSNEEFTHLILEALDGFIMVFSSNGRIYYVSESVTSLLGYLPNELESTTIYDITYQEDQPHLYNILLNPSGARDRHPVKKEDQISFACHIKRGGLDFREDSVYELVQFIGYFRSAVDNDVDNLLASPKFSNTSGSDNKLVFVGTGRLQTPQLIRELSVTDNAKSEFTSRHSLEWKFLFLDHRAPPIIGYLPFEVLGTSGYDYYHVDDLDNVVTCHESLMQKGEGTSCYYRFLTKGQQWIWLQTRFYITYNQWNSKPEFIVCTHYVVSYVGVMKELRAETGGYDKTQSQDVLLPVKQVTASCQTPLAQWPSKSSKTSKSITVGSNLRHRGDGSNTSSISVSLRSSPQSQITHGSCSNAASASSSVVSKPITSSVVTAGFATPAAPILSTVPTHEILHQQGIVMTPAQNQIQDELQRKHEELQQLIVHQQEELRRVSEQLLIARYGILTPLLNAGIPYNPSNPCQQANRCNANNTNVQLPASNGVQNVNVLPIPITVPVPLVPTELFSHPLQICPQQAEIIYGNMETSSSQHQRSAQN

>gi|307200791|gb|EFN80844.1| Aryl hydrocarbon receptor repressor [Harpegnathos saltator]

MNVTILKPPQKDGVTKSNPSKRHRERLNAELDTLASLLPFEQNILSKLDRLSILRLSVSYLRTKSYFQGSGSGNVFVYNFSHLLI

>gi|307198404|gb|EFN79346.1| Single-minded-like protein 1 [Harpegnathos saltator]

MKGGIRSSWLVVCKTKVWQVYAPAKSVEVHRPFLIGGCSTTDLLNSLYLADNYYYQEHSQNEGDKNNNNKDFIKSASGPQEKEMKEKSKNAARSRRVKENQEFLELAKLLPLPAAITTQLDKASIIRLTTSYLKMRAVFPHGLGDEWGAAPPPNNPLESAIKELGSHLLQTLDGFIFVVAPDGKIMYISETASVHLGLSQVELTGNSIFEYIYPDDRNEMISMLNLPSNSPDHRFTYPPPNSRGEIELERAFLLRMKCILAKRNAGLVSEGYKVIHCSGYLKCVFDGPVEYEDSLTRNVGLIRTVGLLAVGHSLPTSSITEIKLHQNMFMFRASLDLKLIFLDARVAHLTGYDPPDLIEKTLYHYVHAADVVHLRQAHRLLLCKGQVTTRYYRFLTKNGGWVWMQSYVTIVHNSRSSRPHCIVSVNYVLTAAENAGLILNCEQKLPCSSPGNPPSAPLSTPAVGANDLDNHSPSPPSYRAGRTKEPPDTDYTDSSGYHNSDYIATGATNHNHYLSPSPYAPHSVDGAAHGEDAYYNPDMFYQFNDPANLQHQQEAQQHLLHHAPSLTTIQHGTQNNQHKRPHSQHLLHHATSLTTIEQQQQQHSPQSGQQKRPYSTSSSSCGSSDGLETHFASSVNLVPSSGYHPSHHHHHHHHHHHMSDTTLNEAGGVIMYPNCGFNNNDNYSVAAAAAATLQHHESYQTQPHNSNGNATVKHPQHPLEASSSAGYTSVIVDSQQYSPNTVQDLHTHQTTPVDDGTPPLHHQHHYETHHQFVR

>gi|307202142|gb|EFN81642.1| Protein trachealess [Harpegnathos saltator]

MLPYQVAMDYGGFQRQSPVGVGVGVGVGVGGPHQGAAGALNINPAFTHSCILELRKEKSRDAARSRRGKENFEFYELAKMLPLPAAITSQLDKASIIRLTISYLKLRDFSSHGDPPWSRDGPPPIKSVKGANRVRSSSSAAIDHFEIHQGTHILQSLDGFAMAVAADGRFLYISETVSIYLGLSQVEMTGSSVFDYIHQQDHAEVAEQLGLGLASSTSTSGPHSSNSGLASPSSAASEEHGSTSTANPDVSSIMSLSASGPYKGYDRAFCVRMKSTLTKRGCHFKSSGYRVVLLLCRLRPQLIFSHTRKTAPPLMGMIGLAIALPPPSVHEIRLETDMFVTRINFDFRIAHCEPKVSELLDYTADELTGKNLYTLCHGEDANRLRKSHIDLIHKGQVLTHYYRLMNKSGGYTWLQTCATVVCNSKNAEEQNIICVNYVISGREYENLIMDCCQLEDGVTGVKREDTAGNDPENGSPDADRGEGRSRGGPTNQHQEQSHRSPAEEREESHGSESEKRGSRHHDNIAQLQQESQTPVGNISTLVVRLRGHKRKIPDDDSSSNDEDDEEDASTTKETSNERSRALSPAASTHSISTTEQTLCSTSPKSRRTEAAGRLDNSESTGTSVKDLEQAMSKHLPVSSLNKSNSPAALHQPTDFSTDALLKQQQQRSTIQWIGAHHHLGHLSPQQSTAAPLPASALLRQLYANRESVIRANVHGITSSGGARAPSSAGIYYPGDGGPNGPLPTPPGSEGSSTYGEHQFVLAAHNQKGTSGGSCADAFTSLVSSYTTATAGGYSVDYHSAMTPPSSVSPRDKQQQQQQQQQQQQQQQQQQQQQQLHPVNVISSYESSSAYAEPVLRHQYEPAQPLPLKPQVYSATVHPSALDAAAAYASAGLAEQPQFYHHPAGSAGFHIYHPTNKSTANGWYTSAS

>gi|307192541|gb|EFN75729.1| Hypoxia-inducible factor 1 alpha [Harpegnathos saltator]

MNARLQEETRDEGEGGDKELGTIAGGGRGAGGNMALGDERQQQGRCTDSLNWIGKASGYADFAEPRRGQTIGLSPESSYASATRPDDDTRVLPADVAAPSSSVTSQLAGDNGPAPRARDIETPRWAPARLPLSASCNLACNFAPAACNNVVPLMTEQALFRDVDDGQSAGPHPSDTCAFAWRNCPQLLPGPVDVEDLAIYFDPSADNGKPPTSGRAIYSRRPRYGQGAARMLLEQPRSQEPRCERLDVASASDLMEPYYNSSRFSYPAEAGDPAFDALPRPCEVGPSDPTPTSTSTPTPTPRSCALQPDFYGQQTTTCEPRLACAAATRELDIRELNRRNLEHDGAPAAPADDWSCYHCPELFNFCSEQRWLQCPASDRCPLYDNSYDHGAPLSPPCLYENLYEGYDGNDNWRYDYAEDEQLLEDYLSNEKRKERSRDAARYRRSRETDIFADLAAVLPVAPQQAAHLDKASVMRLAIAYLKVRAVVDSIPASVTKSESSTEMDDLFPKALNGFMLVLSSDGNMVYLSENVSDYLGVSQMDMMGQSVYEYSHPCDHDELRECLSSKLSEKNGKRVYSFFLRLKCTLTSKGRKVNLKSASYKVIHCTGRLTTIRDSNSNSMEVDNEEGEKEDEEAEREANTSLVLVGNPIPHPSNIEIPLGRHTFLSKHNLSMKFTYADEKLAEYLGWDSEDLMGRSVFEFYHALDNLALDKCFKCLFSKGQCETVAYRFLGKRGGYAWVVTQATLIHCSKQQKPISVVCVNYILR

>gi|307212674|gb|EFN88377.1| Aryl hydrocarbon receptor nuclear translocator-like protein 1 [Harpegnathos saltator]

RELQQQQQQQQQQQQQPQPQPQHSPEPEPELDDLLRFRGSTGQEMVVQEMAQGYTQLLHTAQEPQEFISLDELPRVHQEHHRVHDTCAQIASSASQLYQYHQPQQRGSHQQQQQQEQRNYYSLSTVQESSCPTVYFGEIGTGSGVGELTAASGGGGEGFAPTPTPASNSSSNTATPSSPSATTTTTSSTTTTSTTTATTTATSTSTATIPQVATGAEPPQHMETPPTMVPDQAPASGHHYHHHHHHHHHHHRHHRSTSTTPSHGPATDISDEYAPVSRKRKLSCHVGSDLLDDLGDDAKSVRTNDDSKKQNHSEIEKRRRDKMNTYITELSAMVPMCHAMSRKLDKLTVLRMAVQHLKTILGAVTSYTEGHYKPAFLSDQELKTLILQAAEGFVFVVGCDRGRILYVSESVSQTLNYSQGDLLGQSWFDILHPKDVAKVKEQLSSSDLSPRERLIDAKTMLPVKTDVPQGVSRLCPGARRSFFCRMKRKVEGVRCGEMQVKEEADTTTGCHRRKKQQNVDWKYCVIQCTGYLKSWAPAKIGLEEQEGEADGEACNLSCLVAVGRIQTAISTAALPSRKPHLRPIQFVSRHAMDGKFLFVDQRATPVLGFLPQELLGTSMYEYYHHDDIPHLAESHKAALQTTERVTTQIYRFRSKGANFVRLQSEWKSFRNPWTKDIEYLIAKNSAIFCDMRPGGNNGSEDSSVQGNYDYFTQSNGGLERLISSHVEVSKIGRQIAEEVLDLQRRGEDSSSGSSPGPTTEPGLLNTEMQITTTASPERIPDVSQSGIGSDNSSSNPTSTFNHVANNVQLNSIANDQGASPDEEMMDMIGGTAINESPNPIPSDGNDEAAMAVIMSLLEADAGLGGPVDFSGLPWPLP

>gi|307204506|gb|EFN83186.1| Protein extra-macrochaetae [Harpegnathos saltator]

MKAMVVSPVGGRVPPNRGVLHNGLGIGGTRRDLEAEEVAAYLTKLRSLVPDMPRKRKLSKLEVIQRVIEYICDLQTTLEETNAAQQDTTTTTTTTTTMQSSRQPLEPLLNAAVTTGSTASTTSTATTTTSITAAATIAER

>gi|307204319|gb|EFN83075.1| Hairy/enhancer-of-split related with YRPW motif protein [Harpegnathos saltator]

MWRVVVARSDDNSTLLSSEITSGAVAPGHLPSLPAPPSGHHWGYPPPPHHPPYQPTQHPDMRHHHHHHHHHHDMRPPAPHELRHAAGDPRSQELRHPEMQRHQELPRHQDIHHRPDVARHQDMPGMRADMPEMRSSHEYLELKIDTELRAPENSQPQPQQQSQQQQPQQSQPQQQSQSQQSQVHQQRNLKRAMSDSDCDDVFSEESGKEPCNSPGGDSCQHASRKRRRGMIEKKRRDRINASLGELRRLVPAAARDPHSGKLEKAEILQLTVEHLRTLRNKGPEGYDSTKLAMDYHAVGWGECAAEVGRYLVTMEGLDERDPLRLRLLSHLQSFHREHPPSGAVPSAVPSATTLTSSSTASGYEPPSTVSSGMPAGPGSMQHLLSSSTIGWSQYPGQYPQQQHGKPYRPWGTELAY

>gi|307213739|gb|EFN89077.1| Hairy/enhancer-of-split related with YRPW motif protein 2 [Harpegnathos saltator]

MVTHSMDNILNMQYYPANSHVDASVHSPPPRKRRCLNKEQDPMSHRIIEKRRRDRMNNCLADLSRLIPAEYLKKGRGRVEKTEIIEMAIRHMKHLQGLRQDTKHSPVTPVHAHPEDSVDSMSHSTAASSAAEHYRLGFQECLSETMHFLVEVEGYFARDSLCVQLINHLQQHCDKILATSDRLGFPHHELPVSNGKAINGGGYVHTSIPTIICQPNGHSDHGSSSSGVSSFGDPERPLRPALVPPPTIVSDDSNHSNHSHSTPLMAASCRPANYKFKSSIKQRFSAERVKSSSSPPVVGLAGGCGLDKPMSSPSSSHGVPIFALHDAGSFYVPLTVEAALIRPHLSFIPDTGPDTVLHPVTISVNFNQSMPPAWSQHHSPTHQHQT

>gi|307196842|gb|EFN78278.1| Protein hairy [Harpegnathos saltator]

MPTGGVTVGATPPAQHVPQEAGQPPAQTTPPVTTTRRSSENRRSNKPIMEKRRRARINNSLNDLKTLVLDAMKKDPSRHSKLEKADILEMAVKHMENLQRQSVALTASADPTILNKFRAGFTECASEVGKFPGLDTSVKRRLLAHLASCIGPADANSNNGQTTAQQPVQPAPPTTQLQVHILPQVDATPRIQVQQSNGIFFTNANGTGLQLLPTRLANGDIALVLPAGAKATPVTSPSSSPTPTSPLPTLIPIPQRTASTASSSSSSSSTSSTSTSAASPVTFEASPASFRDQSTGYSSGNSHRDVATSPANGYTSDLEFDPRICSPPLQKPLALVMRKSVVQPVEGKPWRPW

>gi|307196837|gb|EFN78273.1| Protein deadpan [Harpegnathos saltator]

MVDYHSYTMLDEEEFKPRSQSEMTKAELRKSNKPIMEKRRRARINQSLDELKALVLDAMKKDPTRHSKLEKADILEMAVKHIQTVHRQQLSAAIATDPAVLTKFRSGFSECATEVSRYVSQLENVDPLVKQRLVSHLNSCVSNLQQMAPFYSHYVPYMPERLYPEVKVGFQSDFQNGDENNNGSARIQIPNGVQLIPSRLPTGELALLVPQSANISANFPFFPPAADTSARIGQSSAFTAVQRPHSPLPSPSTSTSSYGDESHHSEHYPQSLSPNHHHHQPQRRFKLPEQSPASSSKSFSSPETQKPQISSTSDRKSPITFVESKAADNNVASVKKDPTEEPATRVNSLAATHSLRQPLSVITDKITYNRVPNASALPQLDDLRKRQSDGFLAVSNKKPRYQEATSSTSSSSALSNADVLQNSNEQAAAAEADRESRDRPVTQDSNLNSDKFSMSLAGPSGANGDMWRPW

>gi|307198205|gb|EFN79220.1| Transcription factor HES-4 [Harpegnathos saltator]

MTARLHSLRHQEKKSAGSSHRQHHQHSQHQQAVHQGPSPAPSPSPSLSPSPKHSDGRRANKPLMEKRRRARINQSLAALKALILDSARLENTKHSKLEKADILELTVRHLQRQRSLAQPGLSRYKAGYQDCSREVSRYLDAPDIITGNTTPMDPAVKQRLLRHLDSCVSELDLDLGSRPDSGLGSSPGSVTDRVAGATSPGPLDHHVGGPVGPHCSTAATLTPAGLIKSEIPEMEAARPDSSTTAGDENNNSSRPTSAFSQVNPAALDPHHPHHPAGLPVDHQASTSQQNPNMLSVVQVIPSRLPDGQVVFLLPSHYVQLAAAAAANGISIGPNPPTAIWAATNMSLLKATDKLAKRPHEDLAQEWIQDPTGLKPSKSPRMEQPEQPLDFTTTSKKMKTGSRSTTHLPAVTDQQQQQQHHLRQQQQQQQQQTSVRLPAEGAGSIVEDRPSSHADSEVTVGMPSPSPVGQQPVKDEEGMWRPW

>gi|307212049|gb|EFN87932.1| Enhancer of split mgamma protein [Harpegnathos saltator]

MPLIMSPVTTGIDNCLASPRLLSTTVCKITKPLLERKRRARINRCLDELKNIMVDALETERENISKLEKADILELTVRHLQRLQAARPSGLATAGDDGISAENRWQRGFGHCAAEACRFLSSLSGEAAGRLARHLASGLQTSRQSSSPPPKINLLSPGLTNRIVSNESSSCTVDVSGEVDSEINRANVSPITSTSRADNKHGDEGHGTEPSTTVVFATQCRASMSPVNDKEKLTASCSAKLTKDQRETAIATATTTKSKSCNSSQDDVNAEDDEEIDVERVEERNTIWRPW

>gi|307212041|gb|EFN87924.1| Enhancer of split mgamma protein [Harpegnathos saltator]

MQMHEQIIVVEGQEQPISRTYQYRKVMKPMLERKRRARINRCLDELKELMVTALAGDGENVAKLEKADILELTVRHLHKLQRQQRLSANPVIDADRFRAGYTHCANEVSRCLAATPGVDVALGTKLMTHLGHKLNSMDKTGPLTIHVAAPQSSPSSASELSSDEYAMPLTPASSQPSPVRTDIEAAVSQSHQGLLQVAKPNEPIWRPW

>gi|307212047|gb|EFN87930.1| Enhancer of split mgamma protein [Harpegnathos saltator]

MAPHTSYTPISGMEYEEPVSRTYQYRKVMKPMLERKRRARINRCLDELKDLMVTALQAEGENVAKLEKADILELTVRHLHTLRAARRLTLTPENSYADRFRDGFTQCAQEVSTFLSTSVAAAVHPAAGAQLMRHLGGCLRRLEGPAGANHATATTTSSSTKSSQPTQPNGSQKSVPQNVYSPPQSPVSVASSSVESSDSSNLAVWRPW

>gi|307198173|gb|EFN79194.1| Transcription factor collier [Harpegnathos saltator]

MRRFQVVISTQVGVEGPLLAVSDNMFVHNNSKHGRRAKRLDPSDPGEYNSLYPPIPLQTPCIKAISPNEGWTAGGSTVIIIGENFFDGLQVVFGTMLVWSELITPNAIRVQTPPRQIPGVVEVTLSYKSKQFSLNEPTIDYGFQRLQKLIPRHPGDPEKLPKEIILKRAADLAEALYSMPRSGNTGITGAPRSPGSVHAPAPPTSSSATAFNSYTGQLAVTVQENGSAAKWTDGMYGTVFSTMLSYILDI
