# Supplementary material for: A genome-wide identification and analysis of the basic helix-loop-helix transcription factors in the ponerine ant, Harpegnathos saltator
Source: BMC Evol Biol. 2012 Aug 31;12:165. doi: 10.1186/1471-2148-12-165 (PMC3527142; doi:10.1186/1471-2148-12-165)
Supplement: Additional file 4 — The bHLH sequences belonging to a particular family from 6 insect species, named ponerine ant, red flour beetle, honey bee, domestic silkworm, pea aphid and fruit fly. [file 1471-2148-12-165-S4.doc]

ASCa family

>*PaAse1*

SVARRNARERNRVKQVNNGFATLRQHIPQSVAQSLGSNTAGTHGGSRAGSKKLSKVETLRMAVEYIRSLK

>*PaAse2*

AVARRNARERNRVKQVNNGFATLRQHIPSHIAAGYGDRGKKLSKVETLRMAVEYIRGLQ

>*Tc8*

VVARRNARERRRVQAVNSAFARLRKVVPLENTRGKRVSKVKTLQQAIEYIQALV

>*Tc44*

AVARRNARERNRVKQVNNGFANLRQHIPNFIAAAFESNSRGGNKKLSKVETLRMAVEYIRSLE

>*Tc48*

SVARRNARERNRVKQVNNGFATLRQHIPASVAAAFAPQGPSTGRGASKKLSKVETLRLAVEYIRSLK

>*ac*

SVIRRNARERNRVKQVNNGFSQLDLSNGRRGIGPGANKKLSKVSTLKMAVEYIRRLQ

>*sc*

SVQRRNARERNRVKQVNNSFARLRDLTKGGGRGPHKKISKVDTLRIAVEYIRSLQ

>*l’sc*

SVARRNARERNRVKQVNNGFVNLRQHLPQTVVNSLSNGGRGSSKKLSKVDTLRIAVEYIRGLQ

>*ase*

AVARRNARERNRVKQVNNGFALLREKIPEEVSEAFEAQGAGRGASKKLSKVETLRMAVEYIRSLE

>*BmASCa1*

AVARRNARERNRVRQVNDGFAALRRHIPEEVAAAFETTNSNRGPNKKLSKVETLRMAVEYIRNLE

>*BmASCa2*

SIARRNARERNRVKQVNDGFNALRRHLPASVVAALSGGARRGSSGKKLSKVDTLRMVVEYIRYLQ

>*BmASCa3*

SIARRNARERNRVKQVNDGFNALRKRLPAAVVAALSGGARRGSGKKLSKVDTLRMVVEYIRYLQ

>*BmASCa4*

SVARRNARERNRVKQVNNGFAALRQHIPSAVTAALAGGRGSSRKLSKVDTLRLAVEYIKSLK

>*AmAse1*

AVARRNARERNRVKQVNNGFATLRQHIPSHIAAGYGDRGKKLSKVETLRMAVEYIRGLQ

>*AmAse2*

SVARRNARERNRVKQVNNGFATLRQHIPQSVAQALGGSTAGTHGGSRAGSKKLSKVETLRMAVEYIRSLQ

>*da*(outgroup)

RRQANNARERIRIRDINEALKELGRMCMTHLKSDKPQTKLGILNMAVEVIMTLE

E12/E47 family

>*PaDa*

RRQANNARERIRIRDINEALKELGRMCMTHLKTDKPQTKLGILNMAVEVIMTLE

>*ApDa*

RRQANNARERIRIRDINEALKELGRMCMTHLKTDKPQTKLGILNMAVEVIMSLE

>*Tc50*

RRQANNARERIRIRDINEALKELGRMCMAHLKTDKPQTKLGILNMAVEVIMTLE

>*da*

RRQANNARERIRIRDINEALKELGRMCMTHLKSDKPQTKLGILNMAVEVIMTLE

>*BmE12E47*

PHIYRIRIRDINEALKELGRMCMTHLKSDKPQTKLGILNMAVEVIMTLE

>*AmDa*

FSLISLPPFRIRIRDINEALKELGRMCMTHLKTDKPQTKLGILNMAVEVIMTLE

>*nau*(outgroup)

RRKAATMRERRRLRKVNEAFEILKRRTSSNPNQRLPKVEILRNAIEYIESLE

MyoD family

>*PaNau*

RRKAATLRERRRLRKVNEAFEVLKRRTSNNPNQRLPKVEILRNAIEYIESLE

>*Tc33*

RRKAATLRERRRLRKVNEAFEVLKRRTCNNPGQRLPKVEILRSAIEYIEYLE

>*nau*

RRKAATMRERRRLRKVNEAFEILKRRTSSNPNQRLPKVEILRNAIEYIESLE

>*BmMyoD*

RRKAATLRERRRLRKVNAAFEELRVRARAGSGRLPKLEILRAAIQHIERLQ

>*AmNau*

RRKAATLRERRRLRKVNEAFEILKRRTSNNPNQRLPKVEILRNAIEYIEGLE

>*da*(outgroup)

RRQANNARERIRIRDINEALKELGRMCMTHLKSDKPQTKLGILNMAVEVIMTLE

Ngn family

>*PaTap(bp)*

RRIKANDRERHRMHTLNDALERLRMALPTFPEDTKLTKIETLRFAHNYIWALS

>*Tc46*

RRLKANDRERNRMHMLNEALDRLRCVLPTFPEDTKLTKIETLRFAHSYIFALT

>*ApTap*

RRMKANDRERNRMHMLNEALDRLRCVLPTYPDDAKLTKIETLRFAHNYIWALS

>*tap*

RRMKANDRERNRMHNLNDALEKLRVTLPSLPEETKLTKIEILRFAHNYIFALE

>*BmNgn*

RRMKANDRERNRMHMLNEALDRLRCVLPTFPEDTKLTKIETLRFAHNYIFALS

>*AmTap*

RRIKANDRERHRMHTLNDALERLRMALPTFPEDTKLTKIETLRFAHNYIWALS

>*ac*(outgroup)

SVIRRNARERNRVKQVNNGFSQLDLSNGRRGIGPGANKKLSKVSTLKMAVEYIRRLQ

Mist and Beta3 families

>*PaMistr1*

RRLESNKRERMRMHSLNDAFQSLREVIPHVTKGRRLSKIETLTLAKNYIVALT

>*PaMistr2*

RRLESNERERMRMHSLNDAFEQLREVIPHVKMERKLSKIETLTLAKNYIMALT

>*ApMistr1*

RRLESNERERLRMHSINDAFQSLREVIPHVKKDRRLSKIETLTLAKNYIIALT

>*ApMistr2*

RRLESNERERLRMHSLNDAFEKLREVVPHVKMGRKLSKLETLTLAKNYIMALT

>*Tc45*

LRRLESNERERMRMHSLNDAFEQLREVIPHIKMERKLSKIETLTLAKNYIMALT

>*Mistr*

RRLESNERERMRMHSLNDAFQSLREVIPHVEMERRLSKIETLTLAKNYIINLT

>*BmMist*

RRLESNERERMRMHSLNRAFEDLRRVIPHVKKDNRSLSKIETLTLAKNYVKALT

>*AmMistr1*

RRLESNERERMRMHSLNDAFQSLREVIPHVSKERRLSKIETLTLAKNYIVALT

>*AmMistr2*

RRLESNERERMRMHSLNDAFEQLREVIPHVKMERKLSKIETLTLAKNYIMALT

>*PaOli*

VRLNINARERRRMHDLNDALDELRSVIPYAHSPSVRKLSKIATLLLAKNYILMQG

>*ApOli*

VRLNINARERRRMHDLNDALDELRSVIPYAHSPSVRKLSKIATLLLAKNYILMQA

>*Tc38*

VRLNINARERRRMHDLNDALDELRAVIPYAHSPSVRKLSKIATLLLAKNYILMQA

>*Oli*

VRLNINARERRRMHDLNDALDELRSVIPYAHSPSVRKLSKIATLLLAKNYILMQQ

>*BmBeta3*

VRLNINARERRRMHDLNDALDELRGVIPYAHSPSVRKLSKIATLLLAKNYIMMQA

>*AmOli*

MRLNINARERRRMHDLNDALDELRSVIPYAHSPSVRKLSKIATLLLAKNYILMQG

>*tap*(outgroup)

RRMKANDRERNRMHNLNDALEKLRVTLPSLPEETKLTKIEILRFAHNYIFALE

Atonal family

>*PaCato*

RRLAANARERRRMNGLNDAFDKLREVVPNLGTDHKLSKFETLQMAQSYIAALC

>*PaAto*

RRLAANARERRRMQNLNKAFDRLRTYLPSLGNDRQLSKYETLQMAQSYITALY

>*PaAmos*

RRLAANARERRRMNSLNDAFDRLRDVVPSLGNDRKLSKFETLQMAQTYIAALY

>*ApCato*

RRLAANARERRRMNGLNEAFDRLREAIPTSIEDEHKLSKYETLQMAQSYISALC

>*ApAto*

RRLAANARERRRMQNLNKAFDRLRTVLPTLGNDRQLSKYETLQMAQTYITALY

>*ApAtonal1*

RRLAANARERRRMNGLNEAFDRLRGVVPAADDERKLSKYETLQMAQTYIVALH

>*Tc24*

RRLAANARERRRMNSLNDAFDRLRDVVPSLGNDRKLSKFETLQMAQTYIAALH

>*Tc30*

RRLAANARERRRMQNLNQAFDRLRTFLPQLGQDRQLSKYETLQMAQTYITALY

>*Tc32*

RRLAANARERRRMNGLNEAFDRLRQVIPSLDADHKLSKFETLQMAQTYIAALR

>*cato*

RRQAANARERKRMNGLNAAFERLREVVPAPSIDQKLSKFETLQMAQSYILALC

>*ato*

RRLAANARERRRMQNLNQAFDRLRQYLPCLGNDRQLSKHETLQMAQTYISALG

>*amos*

RRLAANARERRRMNSLNDAFDKLRDVVPSLGHDRRLSKYETLQMAQAYIGDLV

>*BmAtonal*

RRLAANARERRRMQNLNKAFDRLRGHLPSLGADRQLSKYETLQMAQTYIAALY

>*AmAmos2*

RRLAANARERRRMNGLNDAFDKLREVVPSLGADHKLSKFETLQMAQTYIAALC

>*AmAmos1*

RRLAANARERRRMNSLNDAFDRLRDVVPSLGNDRKLSKFETLQMAQTYIAALY

>*AmAto*

RRLAANARERRRMQNLNKAFDRLRAYLPSLGNDRQLSKYETLQMAQSYITALY

>*twi*(outgroup)

QRVMANVRERQRTQSLNDAFKSLQQIIPTLPSDKLSKIQTLKLATRYIDFLC

MyoRa family

>*PaMyoR*

PRNAANARERARMRVLSKAFCKLKTTLPWVPSDTKLSKLDTLRLAATYIAHLR

>*ApMyoR*

PRNAANARERARMRVLSKAFGRLKTTLPWVPADTKLSKLDTLRLATTYIAHLS

>*Tc7*

QRNAANARERARMRVLSKAFCRLKTTLPWVPADTKLSKLDTLRLATSYIAHLR

>*MyoR*

QRNAANARERMRMRVLSSAYGRLKTKLPNIPPDTKLSKLDTLRLATLYIKQLI

>*BmMyoRa*

HRNAANARERARMRVLSKAFRLKTTLPWVPADTKLSKLDTLRLAASYIAHLR

>*AmMyoRa*

PRNAANARERARMRVLSKAFCRLKTTLPWVPADTKLSKLDTLRLAATYIAHLR

>*da*(outgroup)

RRQANNARERIRIRDINEALKELGRMCMTHLKSDKPQTKLGILNMAVEVIMTLE

Net family

>*PaNet*

RRIEANARERTRVHTISAAFDTLRRAIPAYSHNQKLSKLSVLRIACSYIVTLT

>*ApNet*

RRIEANARERSRVHTISAAFDTLRATIPSYSRNQKLSKLSTIRIASAYILTLS

>*Tc35*

RRIEANARERTRVHTISAAFDTLRRAIPSYSHNQKLSKLSVLRIACSYIMTLS

>*net*

RRIEANARERTRVHTISAAYETLRQAVPAYASTQKLSKLSVLRVACSYILTLS

>*BmNet*

RRIEANARERTRVHTISAAFDTLRRSVPAYSHNQKLSKLSVLRIACAYIAALS

>*AmNet*

RRIEANARERTRVHTISAAFDTLRRAIPAYSHNQKLSKLSVLRIACSYIMTLG

>*cato*(outgroup)

RRQAANARERKRMNGLNAAFERLREVVPAPSIDQKLSKFETLQMAQSYILALC

Mesp family

>*PaSage*

YKKSACDRERTRMRDMNRAFELLRSKLPICKPAGKKLSKIESLRHAITYIRHLQ

>*ApSage*

YKKSACDRERTRMRDMNKAFDLLRNRLPKSKPPGKKLSKIESLRRAIWLENCKL

>*sage*

YRRTACDRERTRMRDMNRAFDLLRSKLPISKPNGKKYSKIESLRIAINYINHLQ

>*BmMesp*

YKKTACDRERTRMRDMNRAFDLLRSKLPVTKPSKKKYSKIECLRYVVLIKPKSN

>*AmSage*

YKKSACDRERTRMRDMNRAFELLRSKLPICKPPGKKLSKIESLRHAITYIRHLQ

>*cato*(outgroup)

RRQAANARERKRMNGLNAAFERLREVVPAPSIDQKLSKFETLQMAQSYILALC

Twist family

>*Pa**Twi1*

QRVMANVRERQRTQSLNEAFAALRSVIPTLPSDKLSKIQTLKLATKYIEFLH

>*PaTwi2*

QRKMTNAKERQRTRDLNNAYDDLKKAIPFMSSEKMSKIQTLKLATKYILYLQ

>*ApTwi*

QRVMANVRERQRTQSLNEAFASLRKIIPTLPSDKLSKIQTLKLATRYIDFLY

>*Tc43*

IQHQRVMANVRERQRTQSLNEAFASLRKSIPTMPSDKLSKIQTLKLAARYIDFLY

>*twi*

QRVMANVRERQRTQSLNDAFKSLQQIIPTLPSDKLSKIQTLKLATRYIDFLC

>*BmTwist*

QRVMANVRERQRTQSLNEAFASLRQIIPSLPSDKLSKIQTLQLATQYIEFLY

>*AmTwi*

QRVMANVRERQRTQSLNEAFAALRKIIPTLPSDKLSKIQTLKLATRYIDFLF

>*Pxs*(outgroup)

PRQKINARERYRTFNVNSAYEALRNLIPTEPMNRKLSKIEIIRLASSYITHLS

Paraxis family

>*PaPxs*

QRYQANARERDRTHSVNTAFSALRTLIPTEPADRKLSKIETLRLASSYISHLD

>*ApPxs*

IRSGANARERDRTQSVNSAFDVLRAMIPIDPPDRKLSKIETLQLATKYISHLS

>*Tc10*

QRSQANARERDRTHSVNTAFSTLRTLIPTEPKDRKLSKIETLRLASSYISHLG

>*Pxs*

PRQKINARERYRTFNVNSAYEALRNLIPTEPMNRKLSKIEIIRLASSYITHLS

>*BmParaxis*

QLTIAFNISFVSPSSVNMAFNTLRLLIPTEPPDRKLSKIEILRLAGSYITHLD

>*AmPxs*

EFKREIQEIQSSLCSVNTAFSALRTLIPTEPMDRKLSKIETLRLASSYISHLG

>*twi*(outgroup)

QRVMANVRERQRTQSLNDAFKSLQQIIPTLPSDKLSKIQTLKLATRYIDFLC

Hand family

>*PaHand*

RRNTANKKERRRTQSINNAFADLRDCIPNVPADTKLSKIKTLRLAASYIGYLM

>*ApHand*

RRNTANKKERRRTQSINNAFSDLRDCIPNVPSDTKLSKIKTLRLATSYIGYLM

>*Tc29*

RRTTANKKERRRTQSINNAYADLRDCIPNVPPDTKLSKIKTLRLATSYINYLV

>*Hand*

KRNTANKKERRRTQSINNAFSYLREKIPNVPTDTKLSKIKTLKLAILYINYLV

>*BmHand*

RRTTANKKERRRTENINTAFSDLRDCIPNVPPDTKLSKIKTLRLATSYISYLL

>*AmHand*

RRNTANKKERRRTQSINNAFADLRDCIPNVPADTKLSKIKTLRLAASYIGYLM

>*twi*(outgroup)

QRVMANVRERQRTQSLNDAFKSLQQIIPTLPSDKLSKIQTLKLATRYIDFLC

PTFa and PTFb families

>*PaFer1*

QRQAANMRERRRMQNINDAFEGLRAHIPTLPYEKRLSKVDTLKLAIGYINFLN

>*ApFer1*

QRQAANLRERRRMQSINEAFEGLRAHIPTLPYEKRLSKVDTLKLAIGYINFLS

>*Tc6*

QRRAANIRERRRMFNLNEAFDKLRRKVPTFAYEKRLSRIETLRLAITYISFMS

>*Fer1*

QRQAANLRERRRMQSINEAFEGLRTHIPTLPYEKRLSKVDTLKLAISYITFLS

>*BmPTFa*

QRQAANLRERRRMQSINDAFEGLRAHIPTLPYEKRLSKVDTLKLAIGYISFLG

>*AmFer1*

QRQAANMRERRRMQNINDAFEGLRAHIPTLPYEKRLSKVDTLKLAIGYIKFLN

>*PaFer2*

QRHAANIRERKRMLSINSAFDELRVHVPTFPYEKRLSKIDTLRLAIAYIALLR

>*PaFer3*

QRRAANIRERRRMFNLNEAFDKLRRKVPTFAYEKRLSRIETLRLAITYIAFMG

>*ApFer2*

PSQHIIIMNRLVFSSINSAFDELRGHVPTFPYEKRLSKIDTLRLAIAYIALLR

>*ApFer3*

QRRAANIRERRRMYNLNEAFDKLRRKVPTFAYEKRLSRIETLRLAITYIGFMT

>*Tc37*

QRQAANLRERKRMQSINDAFEGLRAHIPTLPYEKRLSKVDTLKLAIGYINFLS

>*Tc49*

QRHAANIRERKRMLRSAIGPTGSINSAFDELRMHVPTFPYEKRLSKIDTLRLAIAYIALLR

>*Fer2*

QRQAANVRERKRIQRINSAFDELRVHVPTFPYEKRLSKIDTLRLAIAYISLLR

>*Fer3*

QRRAANIRERRRMFNLNEAFDKLRRKVPTFAYEKRLSRIETLRLAITYIGFMA

>*BmPTFb*

CCDFSSINSAFDELRVHVPTFPYEKRLSKIDTLRLAIAYIALLR

>*AmFer2*

NEVKQIVSVIAIFSSINSAFDELRVHVPTFPYEKRLSKIDTLRLAIAYIALLR

>*twi*(outgroup)

QRVMANVRERQRTQSLNDAFKSLQQIIPTLPSDKLSKIQTLKLATRYIDFLC

SCL family

>*PaSCL*

RKLFTNSRERWRQQNVSGAFAELRKLVPTHPPDKKLSKNEILRMAIKYISLLS

>*ApSCL*

PCTVTNSRERWRQHNVTGAFAELRKLVPTHPHDKKLSKNEILRMAIKYIRLLS

>*Tc40*

KKIFTNSRERWRQQNVSGAFAELRKLVPTHPPDKKLSKNEILRMAIRYIRLLS

>*SCL*

RKVFTNTRERWRQQNVSGAFAELRKLVPTHPPDKKLSKNEILRSAIKYIKLLT

>*BmSCL*

RKLFTNCRERWRQQNVSGAFAELRRLVPTHPPDKKLSKNEILRMAIRSIIKLF

>*AmSCL*

RKLFTNSRERWRQQNVSGAFAELRKLVPTHPPDKKLSKNEILRMAIK

>*NSCL*(outgroup)

YRTAHATRERIRVEAFNVSFAELRKLLPTLPPDKKLSKIEILKLAICYIAYLN

NSCL family

>*PaNSCL*

YRTAHATRERVRVEAFNLAFAELRKLLPTLPPDKKLSKIEILRLAICYIAYLN

>*ApNSCL*

YRMAHATRERVRVEAFNVAFGELRKLLPTIPPDKKLSKIEILRLAICYIMYLN

>*Tc31*

YRTAHATRERIRVEAFNVAFAELRKLLPTLPPDKKLSKIEILRLAICYIAYLN

>*NSCL*

YRTAHATRERIRVEAFNVSFAELRKLLPTLPPDKKLSKIEILKLAICYIAYLN

>*BmNSCL*

YRTAHATRERIRVEAFNAAFASLRRLLPTLPPDKKLSKIEILRLAICYIAYLN

>*AmNSCL*

PLLLVVRRERVRVEAFNLAFAELRKLLPTLPPDKKLSKIEILRLAICYIAYLN

>*SCL*(outgroup)

RKVFTNTRERWRQQNVSGAFAELRKLVPTHPPDKKLSKNEILRSAIKYIKLLT

SRC family

>*PaTai*

SQMNKCLNEKRRRTQENLYIDELAELISSTDMSSGKTDKCQILQRTVDQVRRQI

>*Tc41*

AQINKCNNEKRRREQENIYIEELAELISANFADMSSLSVKPDKCAILQETVNQIRSIK

>*ApTai*

SQLNKCRNEKRRREQENIYIEELAELISASFADMNSLSVKPDKCAILQETVNQVRAGT

>*AmTai*

VYSNKCLNEKRRRNQENLFIDELAELISATDMSSGKTDKCQILQRTVDQVRRQI

>*tai*

SQINKCNNEKRRREAENGYIEQLSEILTLNKRGDMTSTKPDKAAILNQVVRTYREIC

>*BmSRC*

FSSNKCQNEKERRKLENETINQLEELLGTCLAEVKQPDKNGIVREATRQIQEVL

>*SREBP*(outgroup)

KRSAHNAIERRYRTSINDKINELKNLVVGEQAKLNKSAVLRKSIDKIRDLQ

Myc family

>*PaDm*

RRIQHNTMEKRRRVYMASLFQQLRSLIPHPNPNFKMPKVRILMEAANYCKNLH

>*Tc4*

KRHLHNNMERQRRIDLRNLFNDLKKLIPDISKKQRAAKVLILRGAAQYCRDLQ

>*ApDm*

KRALHNDLEKSRRVETSMLFMNLSMQVSFLDDNRKIPSKLSILRGAKRECDLMM

>*AmDm*

KRSLHNNMERQRRIELRNAFEDLRILVPAVEKKEKAPKVAILRQAAVYCDTLN

>*dm*

KRNQHNDMERQRRIGLKNLFEALKKQIPTIRDKERAPKVNILREAAKLCIQLT

>*BmMyc*

RRSIHNDMERQRRIGLKNLFDELKMQIPATRDKERAPKVVILREAAALCKKLS

>*Max*(outgroup)

KRAHHNALERRRRDHIKESFTNLREAVPTLKGEKASRAQILKKTTECIQTMR

Mnt and Mad families

>*PaMnt1*

TREVHNKLEKNRRAHLKECFELLKRMLPAQDEKKSSNLSILHAANQYIQTLT

>*PaMnt2*

KRNDSVSLYCCRRAHLRTCLEKLKLLVPLGPETSRHTTLGLLTKAKRFIKVNH

>*Tc39*

TREVHNKLEKHRRAHLKECFDVLKKQLPQTQDEKKTSNLSILHSALRCIQSLK

>*ApMnt*

TREVHNKLEKNRRAHLKECFELLKKQVPASQDEKKTSNLSILRSAIRYIQVLR

>*AmMnt*

TREVHNKLEKNRRAHLKECFELLKRQLPSQEEKKSSNLSILHAAIRHIHVSY

>*Mnt*

TREVHNKLEKERRAQLKECYDLLKKVLPMGDEDRKKTSNLTILDTAHKYVNSLS

>*BmMnt*

TREVHNKLEKNRRAHLKECFELLKRQLPATSDDKKTSNLSILGSAIRYIQVWK

>*ApMad*

YRTTNNEVSKHHTRAHLRNCLEKLKEMVPLGHESSRHTTLGLLTKAKRFIKNLE

>*dm*(outgroup)

KRNQHNDMERQRRIGLKNLFEALKKQIPTIRDKERAPKVNILREAAKLCIQLT

Max family

>*PaMax1*

KRAHHNALERKRRDHIKDSFSSLKNAVPTLQAEKAASRAQILKKAAEYIQTMR

>*PaMax2*

KREHHNYLERKRRDDLKMVFFHLKNNVPTILKGKASRAVILTKTIEYIQKMR

>*Tc22*

KRAHHNALERKRRDHIKDSFSSLRDSVPALNGEKASRAQILKKAAEYIVFMR

>*ApMax1*

KRAHHNALERKRRDHIKDSFTSLRDSVPSLQGEKVASRAQILKKAADYIQFMR

>*ApMax2*

SREHHNISERKRRDQIKDNFEILKETIPVLRGDKPVSRAEILRKASEYIEYMK

>*ApMax3*

SRERHNILERKRRDQIKDNIDILKDMIPVLRGDKPATRAEILKKASEYIEYMK

>*AmMax*

KRAHHNALERKRRDHIKDSFSSLRDSVPVLQGEKVASRAQILKKAAEYIQFMR

>*Max*

KRAHHNALERRRRDHIKESFTNLREAVPTLKGEKASRAQILKKTTECIQTMR

>*BmMax*

KRAHHNALERKRRDHIKDSFTSLRESVPALQNEKVVSNRNSEKGVSTK

>*dm*(outgroup)

KRNQHNDMERQRRIGLKNLFEALKKQIPTIRDKERAPKVNILREAAKLCIQLT

USF family

>*PaUSF1*

RRATHNEVERRRRDKINNWIAKLGKIIPECNVTGTTTNSSSNSGGEGKANYETQSKGGILAKACEYIGELR

>*PaUSF2*

RRATHNEVERRRRDKINSWITKLGKLLPDCDQNTNGEGDAKVNFESQSKGGILARACEYITKLK

>*Tc25*

RRATHNEVERRRRDKINNWITKLSKIIPDGQSKGGILAKACEYILELR

>*ApUSF*

RRLTHNEVERRRRDKINQWIMYMSKIIPDCAEENKTNYDNQSKGGILAKACDYINELK

>*AmUSF2*

RRATHNEVERRRRDKINNWIAKLGKIIPECNAAANGSGSGSGEGKANYETQSKGGILSKACEYITELR

>*AmUSF1*

RRVTHNEVERRRRDKINNWISKLGKLLPECEQSTTADGDVKTNFELQSKGGILARACQYITELR

>*USF*

RRATHNEVERRRRDKINSWIFKLKEMLPEASTSPSTSGSTKGGILIKACEYIKSMQ

>*BmUSF*

RRATHNEVERRRRDKINSWITKLAAMVPNSGMPDSASKGGILAKACDHIADLT

>*SREBP*(outgroup)

KRSAHNAIERRYRTSINDKINELKNLVVGEQAKLNKSAVLRKSIDKIRDLQ

MITF family

>*PaMitf*

KKDNHNMIERRRRFNINDRIKELGTLLPKTNDPYYEIVRDVRPNKGTILKSSVEYIKLLK

>*Tc13*

KKDNHNMVERRRRFNINDRIKELGTLLPKNNDPYYEIVRDVRPNKGTILKSSVEYIKCLK

>*AmMITF*

FLLLLFSVERRRRFNINDRIKELGTLLPKTNDPYYEIVRDVRPNKGTILKSSVEYIKLLK

>*Mitf*

KKDNHNMIERRRRFNINDRIKELGTLLPKGSDAFYEVVRDIRPNKGTILKSSVDYIKCLK

>*BmMITF*

NVLPSVQVERRRRFNINDRIKELGTLLPKTNDPFYEVIRDVRPNKGTILKSSVDYIKCLR

>*bmx*(outgroup)

RREAHTQAEQKRRDAIKKGYDSLQELVPRCQPNDSSGYKLSKALILQKSIEYIGYLN

SREBP family

>*PaSREBP*

KRSAHNAIERRYRTSINDKIIELKNIIVGVEAKLNKSAILRKTIDYIRFLQ

>*Tc14*

KRSAHNAIERKYRTSINDKIVELKNIVVGTEAKLNKSGILKKTIEYIRFLQ

>*ApSREBP*

PKSSHNVIERRYRTSINDKIMELKDMILGTEAKLNKSAILKKAIDYIKYLE

>*AmSREBP*

KRSAHNAIERRYRTSINDKIIELKNIIVGVDAKLNKSAILRKTIDYIRYIK

>*SREBP*

KRSAHNAIERRYRTSINDKINELKNLVVGEQAKLNKSAVLRKSIDKIRDLQ

>*BmSREBP*

KRSAHNAIERRYRTSINDRIVELKNMLVGEEAKLNKSAILRKTIEYIKYLR

>*Mitf*(outgroup)

KKDNHNMIERRRRFNINDRIKELGTLLPKGSDAFYEVVRDIRPNKGTILKSSVDYIKCLK

AP4 family

>*PaCrp1*

RREIANSNERRRMQSINAGFQSLRSLLPHHEGEKLSKAAILQQTAEYIYQLE

>*PaCrp2*

LYSKAKTWERDRRKRMNAYFKTLADLLPPHQEGRKRNKVDILIHASNYIKDLH

>*Tc23*

RREIANSNERRRMQSINNGFQSLRSLLPHHEGEKLSKAAILQQTAEYIYSLE

>*ApCrp*

RREIANSNERRRMQSINAGFQNLRTLIPHHEGEKLSKAAILQHTADYIYQLE

>*AmCrp*

ILSKAKTWERDRRKRMNAYFKTLADLLPPHQEGRKRNKVDILIHASKYIKDLH

>*crp*

RREIANSNERRRMQSINAGFQSLRSLLPRHEGEKLSKAAILQQTFQYIVELE

>*BmAP4*

RREIANSNERRRMQSINAGFQALRTLLPRHEGEKLSKAAILQQTAEYIYNLE

>*SREBP*(outgroup)

KRSAHNAIERRYRTSINDKINELKNLVVGEQAKLNKSAVLRKSIDKIRDLQ

MLX and TF4 families

>*PaMLX*

RRVGHIHAEQKRRYNIKNGFDMLHSLIPQLNQNPNTKLSKAAMLQKGADYIRQLR

>*ApMLX*

RRVCHINAEQKRRCNIKNGFDMLNMLIPQINQNPNTKMSKAAMLQKGADYILQLR

>*AmMLX*

RRVGHIHAEQKRRYNIKNGFDMLHSLIPQLNQNPNTKMSKAAMLQKGADYIRQLR

>*Mlx*

RRAGHIHAEQKRRYNIKNGFDTLHALIPQLQLNPNAKLSKAAMLQKGADHIKQLR

>*BmMLX*

PRRTHLHAEQKRRYNIKNGFDTLQALIPHLNVNPGAKISKAAMLQKGAEYIKQLK

>*PaBmx*

RREAHTQAEQKRRDAIKKGYDSLQDLVPTCQHTDSSGYKISKATVLQKSIDYIQFLL

>*Tc27*

RREAHTQAEQKRRDAIKKGYDTLQELVPTCQQTDVSGYKLSKATVLQKSIDYIQYLQ

>*ApBmx1*

RREAHTQAEQKRRDAIKKGYDCLQDLVPTCQQTDSSGYKLSKATVLQKSIDYIQYLL

>*ApBmx2*

RREAHIQSEQKRREAIKQGYNCLYDLVSTYQQTDISGYKLSKATVLQKSIDYIQYSL

>*AmBmx*

RREAHTQAEQKRRDAIKKGYDSLQDLVPTCQHTDSSGYKLSKATVLQKSIDYIQFLL

>*bmx*

RREAHTQAEQKRRDAIKKGYDSLQELVPRCQPNDSSGYKLSKALILQKSIEYIGYLN

>*BmTF4*

RREAHTQAEQKRRDAIKKGYDSLQDLVPTCQQSDASGYKPSKAAVLQKSIDYIQYLL

>*Mitf*(outgroup)

KKDNHNMIERRRRFNINDRIKELGTLLPKGSDAFYEVVRDIRPNKGTILKSSVDYIKCLK

Clock family

>*PaClk1*

KRKSRNLSEKKRRDQFNMLVNELGSMVSANTRKMDKSTVLKSTILFLKNHN

>*PaClk2*

PRASRNMAEKQRRDNLNTNISTMAALLPIVAGSSRRMDKISILRLAAAFLRTQY

>*Tc3*

KRKSRNLSEKKRRDQFNLLVNELSSMVATGSRKMDKSTVLKSTIAFLKNHN

>*Tc28*

SREMRNRAEKMRRDKLNSYIGELATLVPMVARSAKRMDKTSILRLAATHLRIYQ

>*ApClk*

KRKIRNASEKNRRDQFNNLINELNRMLSTTNRKMDKSTVLKTTINYLNKQK

>*ApRst(1)JH*

SRESRNLAEKNRRQKLNKFITDLTELVPLISNSSKKVEKTSVLRLSAAFLRLKR

>*AmClk2*

PRASRNMAEKQRRDNLNTNISAMAALVPTVAESPRKMDKISILRLAANFLRIHY

>*AmClk1*

FRKSRNLSEKKRRDQFNMLVNELGSMVSSNTRKMDKSTVLKSTILFLKNHN

>*BmClock1*

HRRTRNLSEKKRRDQFNMLVNELSSMVSTNNRKMDKSTVLKSTISFLKNHN

>*BmClock2*

PRELRNKAEKQRRDKLNQSIAELASMVPPVVASNKKIDKTGVLRLTAHYLRAHQ

>*BmClock3*

DRASRIIAEKTRRSQYNALIHQMKSLLSDIAHSQRKVDKTSILRHAVNKLRNEH

>*clk*

KRKSRNLSEKKRRDQFNSLVNDLSALISTSSRKMDKSTVLKSTIAFLKNHN

>*Rst1JH*

GREARNLAEKQRRDKLNASIQELATMVPHAAESSRRLDKTAVLRFATHGLRLQY

>*gce*

GREARNRAEKNRRDKLNGSIQELSTMVPHVAESPRRVDKTAVLRFAAHALRLKH

>*cyc*(outgroup)

RKQNHSEIEKRRRDKMNTYINELSSMIPMCFAMQRKLDKLTVLRMAVQHLRGIR

ARNT family

>*PaTgo*

CRENHCEIERRRRNKMTAYITELSDMVPTCSALARKPDKLTILRMAVAHMKALR

>*Tc26*

MRENHCEIERRRRNKMTAYITELSDMVPTCSALARKPDKLTILRMAVAHMKALR

>*ApTgo*

SRENHCEIERRRRNKMTAYITELSDMVPACQSLARKPDKLTILRMAVNHMKSLR

>*AmTgo*

CRENHCEIERRRRNKMTAYITELSDMVPTCSALARKPDKLTILRMAVAHMRNLR

>*BmARNT*

SRENHCEIERRRRNKMTAYITELSDMVPTCSALARKPDKLTILRMAVAHMKALR

>*tgo*

SRENHCEIERRRRNKMTAYITELSDMVPTCSALARKPDKLTILRMAVAHMKALR

>*cyc*(outgroup)

RKQNHSEIEKRRRDKMNTYINELSSMIPMCFAMQRKLDKLTVLRMAVQHLRGIR

Bmal family

>*PaCyc*

KKQNHSEIEKRRRDKMNTYITELSAMVPMCHAMSRKLDKLTVLRMAVQHLKTIL

>*Tc12*

KKQNHSEIEKRRRDKMNTYITELSAMIPMCHAMSRKLDKLTVLRMAVQHLKTIR

>*ApCyc*

KKHNHSEIEKRRRDKMNSYITELASMIPMCHTMPRKLDKLSVLRMAVQHMKTIR

>*AmCyc*

SRQNHSEIEKRRRDKMNTYITELSAMVPMCHAMSRKLDKLTVLRMAVQHLKTIL

>*BmBmal1*

CRQNHSEIEKRRRDKMNTFISELSAMIPMCGAMARKLDKLTVLRMAVQHLRTVR

>*BmBmal2*

CRQNHSEIEKRRRDKMNTFISELSAMIPMCGAMARKLDKLTVLRMDVQHLRTVR

>*cyc*

RKQNHSEIEKRRRDKMNTYINELSSMIPMCFAMQRKLDKLTVLRMAVQHLRGIR

>*tgo*(outgroup)

SRENHCEIERRRRNKMTAYITELSDMVPTCSALARKPDKLTILRMAVAHMKALR

AHR family

>*PaDys1*

ASKSTKGASKLRRDLINAEIANLRDLLPLPPSTRQRLSQLQLMALVCVFLRKAN

>*PaDys2*

KKREHALALAPRIHGKNNARSGLRHTPFSPLSLRLSPAQTLEDAIAFRGARS

>*PaSs*

DGVTKSNPSKRHRERLNAELDTLASLLPFEQNILSKLDRLSILRLSVSYLRTKS

>*Tc42*

DGVTKSNPSKRHRERLNAELDTLASLLPFEQNILSKLDRLSILRLSVSYLRTKS

>*ApDys*

ATKSTKGASKLRRDLINAEIANLRDLLPLPPSTRQRLSQLQLMALVCVYVRKAN

>*ApSs*

GGVGKSNPSKRHRERLNAELDTLANLLPFEHNILSKLDRLSILRLSVSYLRTKS

>*AmSS*

DGVTKSNPSKRHRERLNAELDTLASLLPFEQNILSKLDRLSILRLSVSYLRTKS

>*AmDys*

ASKSTKGASKLRRDLINAEIANLRDLLPLPPSTRQRLSQLQLMALVCVFLRKAN

>*BmAHR1*

DGVTKSNPSKRHRERLNAELDTLASLLPFEQNILSKLDRLSILRLSVSYLRTKS

>*BmAHR2*

PTKSTKGASKMRRDLINAEISNLRDLLPLPPSTRQRLSQLQLMALVCVYVRKMN

>*BmAHR3*

QGKSTKGASKLRRDLINAEIANLRDLLPLPPSTRQRLSQLQLMALVCVYVRKSN

>*dys*

ANKSTKGASKMRRDLINAEIANLRDLLPLPQSTRQRLSQLQLMALVCVYVRKAN

>*ss*

DGVTKSNPSKRHRERLNAELDLLASLLPFEQNILSKLDRLSILRLSVSYLRTKS

>*clk*(outgroup)

KRKSRNLSEKKRRDQFNSLVNDLSALISTSSRKMDKSTVLKSTIAFLKNHN

Sim, Trh and HIF families

>*PaSim*

MKEKSKNAARSRRVKENQEFLELAKLLPLPAAITTQLDKASIIRLTTSYLKMRA

>*ApSim*

MRERSRNAARTRRENENAEFLELAKLLPLPAAITSQLDKASVIRLTTSYLKMRH

>*AmSim*

MKEKSKKAARIRRDRENQEFLELAKLLPVPAALTGQMDKASVIRLTTSYLKMRA

>*BmSim*

MKEKSKNAARSRREKENTEFLELAKLLPLPSAITSQLDKASVIRLTTSYLKMRQ

>*sim*

MKEKSKNAARTRREKENTEFCELAKLLPLPAAITSQLDKASVIRLTTSYLKMRQ

>*PaTrh*

RKEKSRDAARSRRGKENFEFYELAKMLPLPAAITSQLDKASIIRLTISYLKLRD

>*Tc1*

RKEKSRDAARSRRGKENFEFYELAKMLPLPAAITSQLDKASIIRLTISYLKLRD

>*ApTrh*

RKEKSRDAARSRRGKENYEFYELAKMLPLPAAITSQLDKASIIRLTISYLKLRD

>*AmTrh*

RKEKSRDAARSRRGKENFEFYELAKMLPLPAAITSQLDKASIIRLTISYLKLRE

>*BmTrh*

RKEKSRDAARSRRGKENYEFYELAKMLPLPAAITSQLDKASIIRLTISYLKLRD

>*trh*

RKEKSRDAARSRRGKENYEFYELAKMLPLPAAITSQLDKASIIRLTISYLKLRD

>*PaSima*

RKERSRDAARYRRSRETDIFADLAAVLPVAPQQAAHLDKASVMRLAIAYLKVRA

>*Tc2*

RKEKSRDAARSRRSKETEVFTDLGNALPISQEQVSQLDKASVMRLAIAYLRVRD

>*ApSima*

RKEKSRDAARSRRSKETEIFTDLGSALPLPASVISQLDKATVMRLTIASFKIMD

>*AmHIF*

RKEKSRDAARYRRSKETDIFTDLAAALPVTPEQAAHLDKASVMRLAIAYLKVRS

>*BmHIF*

RKEKSRVAARCRRTKEMQIFSELTAALPAKKEEVEQLDKASVMRLAISYLRVRD

>*sima*

RKEKSRDAARCRRSKETEIFMELSAALPLKTDDVNQLDKASVMRITIAFLKIRE

>*dys*(outgroup)

ANKSTKGASKMRRDLINAEIANLRDLLPLPQSTRQRLSQLQLMALVCVYVRKAN

Emc family

>*PaEmc*

TKLRSLVPDMPRKRKLSKLEVIQRVIEYICDLQ

>*Tc11*

SKLKDLVPFMPKNRKLSKLEVIQYVIDYICDLQ

>*ApEmc*

SKLKDLVPLIPKDKRISRLEVIHHVIDYICDLE

>*AmEmc*

TKLRSLVPDMPRKRKLSKLEVIQRVIEYICDLQ

>*BmEmc*

SKLQDLVPFMPKNRKISKLEVIQHVIDYICDLQ

>*emc*

SKLKDLVPFMPKNRKLTKLEIIQHVIDYICDLQ

>*crp*(outgroup)

RREIANSNERRRMQSINAGFQSLRSLLPRHEGEKLSKAAILQQTFQYIVELE

Hey family

>*PaHey*

RKRRRGMIEKKRRDRINASLGELRRLVPAAARDPHSGKLEKAEILQLTVEHLRTLR

>*PaStich1*

DPMSHRIIEKRRRDRMNNCLADLSRLIPAEYLKKGRGRVEKTEIIEMAIRHMKHLQ

>*Tc21*

RKKRRGVIEKKRRDRINMSLSELKRLVPSAFEKQGSAKLEKAEILQMTVDHLKMLH

>*ApHey*

RKKRRGIIEKRRRDRINTSLSELRRLVPTAYEKQGSAKLEKAEILQLTVDHLKMIH

>*ApStich1a*

DPMSHRIIEKRRRDRMNNCLADLSRLIPAEYMKKGRGRVEKTEIIEMAIKHMKYLQ

>*ApStich1b*

APSKNRVYEKERRDRLNVSFEELRTVLPPSDSNASLGKADIINHAIDLIRVLQ

>*AmStichl*

DPMSHRIIEKRRRDRMNNCLADLSRLIPAEYLKKGRGRVEKTEIIEMAIRHMKHLQ

>*AmHey*

RKRRRGMIEKKRRDRINASLGELRRLVPAAARDPHSGKLEKAEILQLTVEHLRTLR

>*BmHey1*

RKRRRGVIEKKRRDRINTSLTELKRLVPAACEKQGSAKLEKAEILQLTVDHLKMLH

>*BmHey2*

DPMSHRIIEKRRRDRMNNCLADLSRLIPPEYLKKGRGRVEKTEIIEMAIRHLKYLQ

>*Hey*

RKKRRGVIEKKRRDRINSSLTELKRLVPSAYEKQGSAKLEKAEILQLTVEHLKSLQ

>*Stich1*

DPLSHRIIEKRRRDRMNSCLADLSRLIPPQYQRKGRGRIEKTEIIEMAIRHLKHLQ

>*Her*(outgroup)

REVFKPMMERKRRSRINRCLDFIKDLLQEVSHLDGETMAKMDMGDVLELAVHHLSKKN

H/E(spl) family

>*PaH1*

RRSNKPIMEKRRRARINNSLNDLKTLVLDAMKKDPSRHSKLEKADILEMAVKHMENLQ

>*PaH2*

RKSNKPIMEKRRRARINQSLDELKALVLDAMKKDPTRHSKLEKADILEMAVKHIQTVH

>*PaSide*

RRANKPLMEKRRRARINQSLAALKALILDSARLENTKHSKLEKADILELTVRHLQRQR

>*PaE(spl)1*

CKITKPLLERKRRARINRCLDELKNIMVDALETERENISKLEKADILELTVRHLQRLQ

>*PaE(spl)*3

RKVMKPMLERKRRARINRCLDELKDLMVTALQAEGENVAKLEKADILELTVRHLHTLR

>*PaE(spl)*2

RKVMKPMLERKRRARINRCLDELKELMVTALAGDGENVAKLEKADILELTVRHLHKLQ

>*Tc15*

RRSNKPIMEKRRRARINNSLNELKTLILDAMKKDPARHSKLEKADILEMTVKHLQNLQ

>*Tc16*

RKVMKPMLERKRRARINRCLDELKELMVTALQSEGENVSKLEKADILELTVRHLHKLR

>*Tc17*

RRANKPLMEKRRRARINQSLAALKTLILDSAKADNTKHSKLEKADILELTVRHFQRHR

>*Tc18*

ERVRKPLMEKKRRARINDSLEALKQILLDSKTTLKESSGKKSGQRTAKLEKADILEMTVRYVQHLR

>*Tc19*

FQTHKPIMEKRRRARINHCLNEIKTLILEAMNKDPARHSKLEKADILEMAVKHLQNVQ

>*Tc20*

DPMSHRIIEKRRRDRMNNCLADLSRLIPTEYLKKGRGRIEKTEIIEMAIKHMKYLQ

>*ApH*

RRSNKPIMEKKRRARINNCLNELKTLILDATKKDPARHSKLEKADILEMTVKHLESMQ

>*ApDpn*

RKNNKPIMEKKRRARINQCLNELKTLILDALKKDPARHTKLEKADILEMTVRHLQSLH

>*ApSide*

KRANKPLMEKRRRARINQSLALLKTLILDSTRTENTKHSKLEKADILELTVRHLQRQK

>*ApHES1*

RKVMKPMLERKRRARINRCLDELKELMVVALQNEGENVSKLEKADILELTVRHLHKLR

>*ApHES2*

RKITKPLLERKRRARINRCLDELKDLMFSALEAEGENVDKLEKADILEFTVKHLQKIT

>*ApHES3*

RKIRKPLMEKKRRARINQSLDELKRIVVDAEKFAGQDLSRVNKLEKADILEMTVRYLKRKS

>*AmSide*

FQANKPLMEKRRRARINQSLAALKALILDSARLENTKHSKLEKADILELTVRHLQRQR

>*AmH*

LQSNKPIMEKRRRARINNCLNDLKTLILDAMKKDPARHSKLEKADILEMTVKHLETLQ

>*AmDpn*

LQSNKPIMEKRRRARINQCLDELKSLILEAMKKDPARHSKLEKADILEMTVKHLQAVQ

>*AmE(spl)2*

RKVMKPMLERKRRARINRCLDELKDLMVTALQAEGENVAKLEKADILELTVRHLHTLR

>*AmE(spl)3*

LQVMKPMLERKRRARINRCLDELKDLMVTALAGDGENVAKLEKADILELTVRHLHKLQ

>*AmE(spl)1*

QQITKPLLERKRRARINKCLDELKNLMIDALETEGEDISKLEKADILELTVRHLQRLQ

>*BmHEspl1*

TNKPIMEKKRRARINRCLNELKDLLLDSEKDPTRHSKLEKADILELTVKHLQKLQ

>*BmHEspl2*

LQSNKPIMEKRRRARINNCLNELKALILDAMKKDPARHSKLEKADILEMTVKHLEGLR

>*BmHEspl3*

RKVMKPMLERKRRARINRCLDELKDLMVTALQAEGENVSKLEKADILELTVRHLHNLK

>*BmHEspl4*

RKVMKPMLERKRRARINRCLDELKELMVSALQSEGENVAKLEKADILELTVRHLHKLR

>*BmHEspl5*

KKITKPLLERKRRARINRCLDELKDLMVGALEIDDDNLSKLEKADILELTVNHLTKLH

>*h*

RRSNKPIMEKRRRARINNCLNELKTLILDATKKDPARHSKLEKADILEKTVKHLQELQ

>*dpn*

RKTNKPIMEKRRRARINHCLNELKSLILEAMKKDPARHTKLEKADILEMTVKHLQSVQ

>*side*

KRTNKPLMEKRRRARINQSLAILKALILESTKTQNAKNGEGQAKHTKLEKADILELTVRHFQRHR

>*Esplm3*

RKVMKPLLERKRRARINKCLDDLKDLMVECLQQEGEHVTRLEKADILELTVDHMRKLK

>*Esplm5*

LKVKKPLLERQRRARMNKCLDTLKTLVAEFQGDDAILRMDKAEMLEAALVFMRKQV

>*Esplm8*

QKVKKPMLERQRRARMNKCLDNLKTLVAELRGDDGILRMDKAEMLESAVIFMRQQK

>*Esplm7*

RKVMKPLLERKRRARINKCLDELKDLMAECVAQTGDAKFEKADILEVTVQHLRKLK

>*EsplmBg*

RKVMKPMLERKRRARINKCLDELKDLMVATLESEGEHVTRLEKADILELTVTHLQKMK

>*EsplmCd*

RKVTKPLLERKRRARMNLYLDELKDLIVDTMDAQGEQVSKLEKADILELTVNYLKAQQ

>*EsplmAb*

RKVMKPMLERKRRARINKCLDELKDIMVECLTQEGEHITRLEKADILELTVEHMKKLR

>*Her*

REVFKPMMERKRRSRINRCLDFIKDLLQEVSHLDGETMAKMDMGDVLELAVHHLSKKN

>*Hey*(outgroup)

RKKRRGVIEKKRRDRINSSLTELKRLVPSAYEKQGSAKLEKAEILQLTVEHLKSLQ

COE family

>*Pakn(col)*

SLNEPTIDYGFQRLQKLIPRHPGDPEKLPKEIILKRAADLAEALY

>*Tc47*

SLNEPTIDYGFQRLQKLIPRHPGDPEKLPKEIILKRAADLAEALY

>*ApKn(col)*

SLSEPTIDYGFQRLQKFVPRYPGDPEKLPKEVILKRAADLAEALY

>*Amkn*

ALNEPTIDYGFQRLQKLIPRHPGDPEKLPKEIILKRAADLAEALY

>*BmCOE*

ALNEPTIDYGFQRLQKLIPRHPGDPEKLPKEIILKRAADLAEALY

>*Kncol*

ALNEPTIDYGFQRLQKLIPRHPGDPEKLQKEIILKRAADLVEALY

>*crp*(outgroup)

RREIANSNERRRMQSINAGFQSLRSLLPRHEGEKLSKAAILQQTFQYIVELE
